# Identification of pathogenic variants in cancer genes using base editing screens with editing efficiency correction

**DOI:** 10.1186/s13059-021-02305-2

**Published:** 2021-03-10

**Authors:** Changcai Huang, Guangyu Li, Jiayu Wu, Junbo Liang, Xiaoyue Wang

**Affiliations:** State Key Laboratory of Medical Molecular Biology, Department of Biochemistry and Molecular Biology, Institute of Basic Medical Sciences Chinese Academy of Medical Sciences, School of Basic Medicine Peking Union Medical College, Beijing, China

## Abstract

**Background:**

Millions of nucleotide variants are identified through cancer genome sequencing and it is clinically important to identify the pathogenic variants among them. By introducing base substitutions at guide RNA target regions in the genome, CRISPR-Cas9-based base editors provide the possibility for evaluating a large number of variants in their genomic context. However, the variability in editing efficiency and the complexity of outcome mapping are two existing problems for assigning guide RNA effects to variants in base editing screens.

**Results:**

To improve the identification of pathogenic variants, we develop a framework to combine base editing screens with sgRNA efficiency and outcome mapping. We apply the method to evaluate more than 9000 variants across all the exons of *BRCA1* and *BRCA2* genes. Our efficiency-corrected scoring model identifies 910 loss-of-function variants for *BRCA1/2*, including 151 variants in the noncoding part of the genes such as the 5′ untranslated regions. Many of them are identified in cancer patients and are reported as “benign/likely benign” or “variants of uncertain significance” by clinicians. Our data suggest a need to re-evaluate their clinical significance, which may be helpful for risk assessment and treatment of breast and ovarian cancer.

**Conclusions:**

Our results suggest that base editing screens with efficiency correction is a powerful strategy to identify pathogenic variants in a high-throughput manner. Applying this strategy to assess variants in both coding and noncoding regions of the genome could have a direct impact on the interpretation of cancer variants.

**Supplementary Information:**

The online version contains supplementary material available at 10.1186/s13059-021-02305-2.

## Introduction

Millions of sequence variants have been identified through genome sequencing of cancer samples over the past decades [1]. The ability for us to interpret the functional impacts of these variants on cancer development remains poor. Pathogenic variants were mostly identified by the association of the variant with disease status, either in families or in a large cohort of people [2]. However, such information on the carriers of the variants is often difficult to collect. As a result, many of the genetic variants are classified as “variants of uncertain significance (VUS)” even though they are in well-known cancer genes. For example, for *BRCA1* and *BRCA2*, two genes that are targets for prevention and therapy of several types of cancers [3], more than half of the 5095 and 8010 single nucleotide variants were classified as VUS or “conflicting interpretations” in the *ClinVar* database as of January 2020 [4].

In vitro functional assays have been used as important supporting evidence for determining the pathogenicity of variants [2], yet the method to introduce mutations into cells has limited their scalability. Most methods utilized exogenous vectors to carry cDNAs with mutations as transgenes while inhibiting or deleting the endogenous copy of the target gene [5–7]. These strategies could only assay mutations in coding regions and the mutations are not tested in their native genomic context. The newly developed CRISPR-Cas9 system is a way to directly introduce mutations in both coding and noncoding regions in the genome [8, 9]. Cas9-sgRNA complex could induce DNA double-strand breaks at target sites, which can be repaired to desired genotype in the presence of a repair template [10]. Saturated mutagenesis using CRISPR-Cas9 system with massive synthetic repair templates has been successfully applied for the functional assessment of all the nucleotide variations in 13 of the 23 exons of *BRCA1* [11]. However, this method requires generation of DNA double-strand breaks, which could induce p53-mediated growth arrest in some cell types [12]. Also, the low ratio of base substitutions to insertion or deletion after the repair [13], and the requirement of synthesis of a large amount of templates make the saturated genome editing only feasible for relatively small loci.

Base editors, which are usually built by fusing a deoxynucleotide deaminase to a nuclease-deficient or nickase Cas protein [14, 15], is an efficient way to generate direct base substitutions throughout the genome without inducing DNA double-strand breaks [16–20]. Applying cytosine base editors in genetic screens has allowed functional assessment of C→T or C→G mutations in human cells and yeast [21, 22]. In these studies, the requirement of a protospacer adjacent motif (PAM) limited the targeting scope of base editing screens. For the canonical SpCas9-based base editors, a PAM sequence of “NGG” 13 to 17 nucleotides downstream the target site is required for efficient base editing. Recent development of the SpCas9 variants has relaxed the PAM requirement to “NGN”, making it possible to evaluate a much larger group of sequence variants in base editing screens.

However, the variability in editing efficiencies poses a challenge for quantitative functional assessment of variants in base editing screens. In a pooled screen, the change of sgRNA abundance between two conditions is often used to evaluate the effects of sgRNAs [23]. Because the activities of sgRNAs to induce an editing event at a target site are highly variable [24, 25], the functional effect of a mutation could be masked by the low editing efficiency of its targeting sgRNA. In addition, several bases could be edited simultaneously in the editing window, but at different frequencies [24, 25], making the interpretation of sgRNA effects difficult.

In order to account for the variability in editing activities, we developed a framework to incorporate editing efficiency correction in base editing screens. We demonstrated that our efficiency correction framework improved the identification of loss-of-function variants from base editing screens. Applying base editing screens with efficiency correction, we assessed functional impacts of C·G→T·A or A·T→G·C conversions for about 9000 sites in *BRCA1* and *BRCA*2 genes, and identified 910 variants that have negative effects on *BRCA1/2* function. These variants include 185 variants that were marked as VUS or “benign/likely benign” in *ClinVar*, suggesting the power of the method for determining the functional significance for previously unknown variants.

## Results

### Base editing screens for loss-of-function variants in *BRCA1* and *BRCA2* in eHAP human haploid cells

**Fig. 1:**
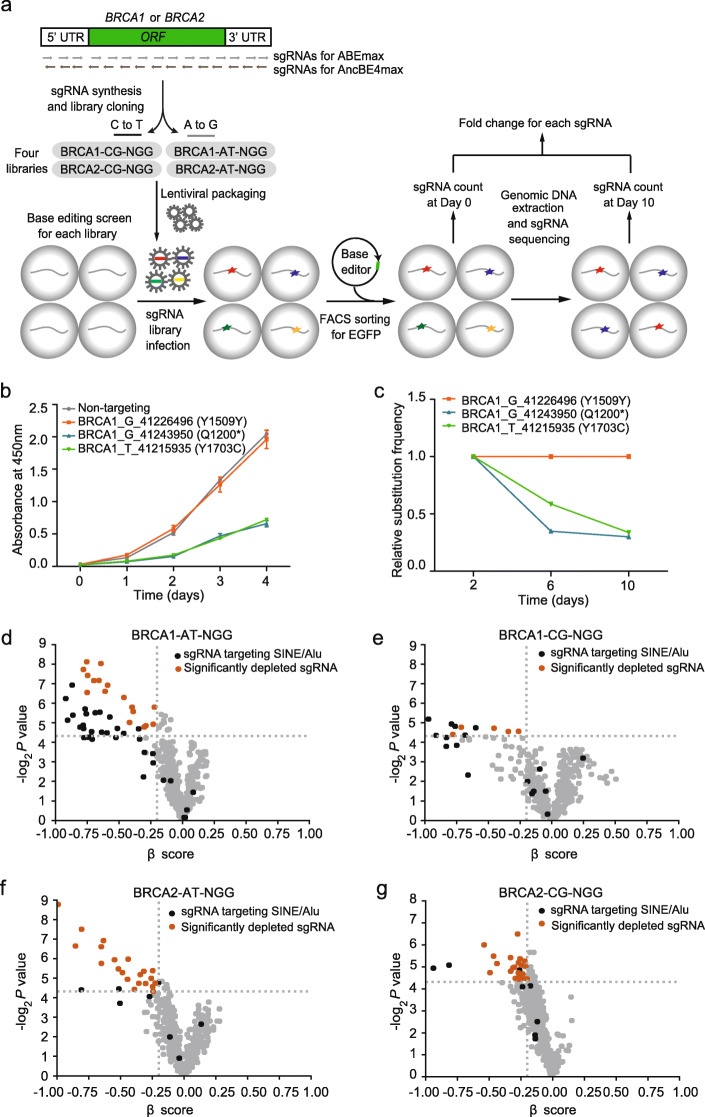
A proof-of-concept screen for pathogenic variants in *BRCA1* and *BRCA2* using cytosine and adenine base editors. **a** Schematic workflow of the base editing screen. sgRNAs targeting all the possible C/G or A/T bases across the exon regions of *BRCA1* and *BRCA2* were cloned and delivered into eHAP cells through lentivirus. After puromycin selection, cells were transfected with base editors and selected by EGFP signals through fluorescence-activated cell sorting. sgRNA cassettes were PCR amplified from cells at day 0 and day 10 and sequenced. **b** Cell viability analysis of eHAP cells after base editing with different sgRNAs using CCK-8 assay. The control group was infected with the sgRNA targeting wild type GFP gene. The three sgRNAs were expected to generate Q1200*, Y1509Y, and Y1703C mutations, respectively. Error bars represent SEMs from three independent experiments. **c** The relative frequency of the substitution variants induced by three sgRNAs, measured at day 0, day 4, and day 8 after base editing. The frequency was normalized to 100% at day 0. **d–g** The essentiality scores (*β* scores) and their *P* values reported by the MAGeCK-MLE algorithm for the four base editing screens: BRCA1-AT-NGG (**d**), BRCA1-CG-NGG (**e**), BRCA2-AT-NGG (**f**), and BRCA2-CG-NGG (**g**). The dashed lines indicate *P* = 0.05. The black dots indicate the sgRNAs targeting the SINE/Alu repeats. The red dots indicate the sgRNAs that were significantly depleted after base editing (fold change < 0.8, *P* < 0.05)

To test the feasibility for identifying functional variants using CRISPR-Cas9 base editing screens, we performed a proof-of-concept screen with sgRNAs targeting 927 bases in *BRCA1* and 981 bases in *BRCA2* in eHAP cells (Fig. 1a). We chose the eHAP cells because they are haploid human cells that could facilitate analysis of loss-of-function alleles. Reducing the expression levels of *BRCA1* or *BRCA2* by RNAi in eHAP cells inhibited the cell growth (Additional file 1: Figure S1a-d; Additional file 2: Table S1), confirming that these two genes are essential in haploid human cells [11, 26].

We selected the cytosine base editor AncBE4max and adenine base editor ABEmax to generate C·G→T·A or A·T→G·C substitutions, respectively. To confirm their abilities in generating nucleotide variants in eHAP cells, three sgRNAs targeting *BRCA1* were used. The sgRNAs were delivered to eHAP cells in lentiviral packaged vectors, followed by transfection of the EGFP-tagged base editors. After 48 h, we performed fluorescence-activated cell sorting (FACS) to enrich EGFP-positive cell populations. We found that all the three sgRNAs generated desired mutations in the endogenous *BRCA1* locus in eHAP cells (Additional file 1: Figure S1e). The sgRNA that could generate a premature stop codon in exon 10 (Q1200*) of *BRCA1* reduced cell viability, while the sgRNA generating only a synonymous variant (Y1509Y) had no impact on cell growth (Fig. 1b). The sgRNA resulting in a mutation in the BRCA1 C terminal (BRCT) domain (Y1703C) also had a negative effect on cell viability (Fig. 1b). Consistent with the effects on cell viability, the relative proportions of the Q1200* and Y1703C variants decreased after 10 days of cell growth, while that of the synonymous mutation did not change (Fig. 1c). These results confirmed the feasibility of the base editing assay and set the basis for high-throughput screens.

For the screen, 1908 sgRNAs targeting *BRCA1/2* were designed and divided in four pools, named *BRCA1*-CG-NGG and *BRCA2*-CG-NGG for cytosine base editing, and *BRCA1*-AT-NGG and *BRCA2*-AT-NGG for adenine base editing (Additional file 3: Table S2). Each library contained about 10% non-targeting sgRNAs as negative controls. For *BRCA1*-CG-NGG and *BRCA2*-CG-NGG libraries, we also included 230 sgRNAs that could generate stop codon in essential genes other than *BRCA1/2* as additional controls for cell lethality in the screen (hereafter referred as “essential gene controls”). To perform the screen, eHAP haploid cells were infected with each of the four lentiviral sgRNA libraries at a multiplicity of infection of 0.3 and transfected with the corresponding base editors (Fig. 1a). We sequenced the sgRNAs on day 10 and day 0 at a median depth over 2000 and calculated the natural logarithm (ln) ratio of their relative abundance on these 2 days (Fig. 1a). We performed each screen twice and we found significant correlations of the ln ratio between replicates (Additional file 1: Figure S2a-d). As expected, the essential gene controls in the C->T library had an average ln ratio of less than 0, indicating that they were more likely to be depleted at day 10 compared to day 0 (Additional file 1: Figure S2e-f). With the adjusted *p* value less than 0.05, 58 sgRNAs for *BRCA1* and 58 sgRNA for *BRCA2* were significantly depleted at day 10 (Fig. 1d–g). Among them, we found 47 sgRNAs that were targeting the SINE/Alu repeats in the 3′ UTR region of *BRCA1/2* [27]. It was reported that base editing on endogenous transposable elements (TEs) could induce cellular toxicity [28]. Therefore, all the sgRNAs targeting more than one site in the genome were excluded from further analysis.

### Mapping editing efficiencies and outcomes of sgRNAs

**Fig. 2:**
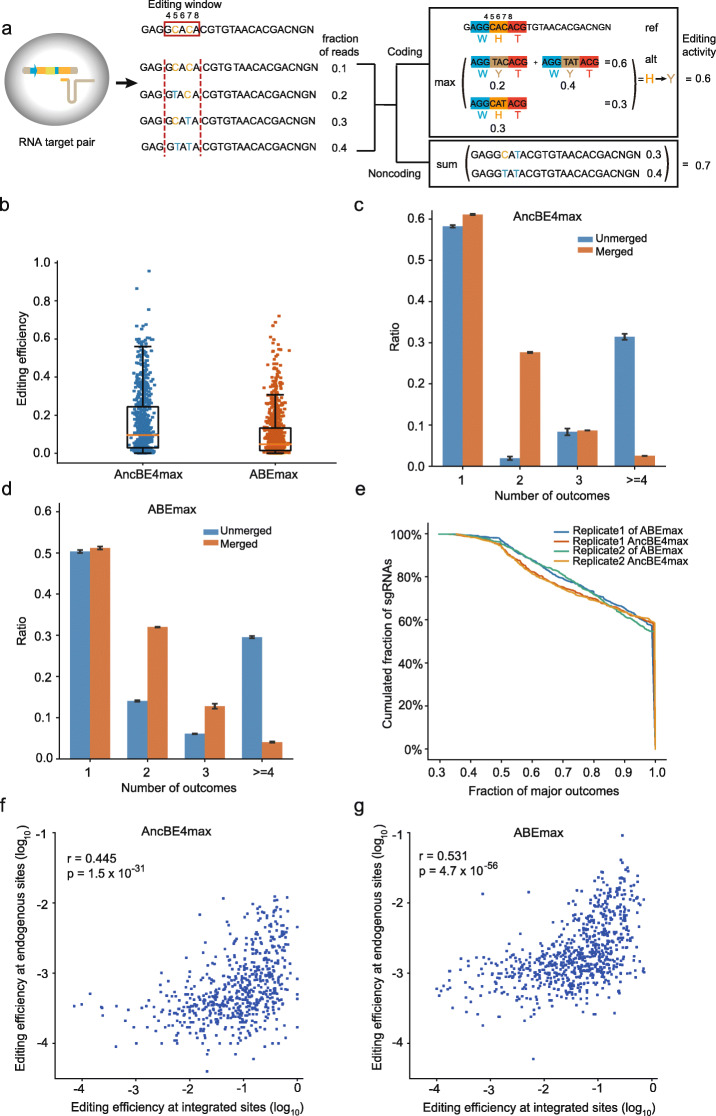
Base editing outcome and efficiency mapping for sgRNAs using genome-integrated target site library assay. **a** Schematic of the outcome and efficiency mapping from edited reads in integrated target sites. For coding regions, the outcome for an sgRNA was determined as the amino acid change with the highest fraction of reads. For noncoding regions, outcome was defined as the single base change of variants with the highest fraction of reads. **b** Editing frequencies at the integrated target sites in cells edited with AncBE4max or ABEmax, respectively. The orange line represents the median of the fraction of edited reads for all sgRNAs in the library. **c**, **d** Fraction of sgRNAs associated with different number of outcomes before and after merging outcomes at amino acid level after editing with AncBE4max (**c**) or ABEmax (**d**). **e** Distribution of the major outcome fraction after editing with AncBE4max and ABEmax. **f**, **g** Correlations of editing efficiencies at integrated target sites measured from the genome-integrated target site library assay and those at endogenous sites determined from amplicon sequencing for cells edited with AncBE4max (**f**) or ABEmax (**g**). Pearson’s correlation coefficients are shown

For one sgRNA, base editors may edit more than one nucleotide in its targeting window, resulting in complex editing outcomes. To associate the effects of sgRNAs to the functional effects of nucleotide variants, we adapted a high-throughput method to measure the editing outcomes for the sgRNAs in our library [29, 30] (Fig. 2a, Additional file 1: Figure S3a, b). In this assay, thousands of sgRNAs and their targeted sequences including the PAM sequences were paired in plasmids and integrated into the genomes of HEK293T cells, and the plasmids containing the base editors were transfected subsequently (Additional file 1: Figure S3a, b; Additional file 5: Table S4). We determined the editing outcomes by sequencing each integrated target sequence at a median depth of more than 4000. For AncBE4max and ABEmax, the median fractions of edited reads were 9.6% and 4.6% (Fig. 2b), respectively. The sgRNAs had high variability on the abilities to modify their targeted sequences, for AncBE4max, the 25% quantile fraction of edited reads was 2.8% and the 75% quantile fraction was 25.4% (Fig. 2b). We also observed various levels of co-editing of nucleotides in the editing window of 4–8 bp for AncBE4max and ABEmax (Additional file 1: Figure S3c, d). For two consecutively Cs, the fraction of reads with both C edited compared to the reads with at least one C-edited were between 22 and 82% (Additional file 1: Figure S3c). For two consecutively As, the fraction of reads with both A edited compared to the reads with at least one A-edited were between 8 and 18% (Additional file 1: Figure S3d). As a result, 41.7% of sgRNAs generated more than one editing products with AncBE4max and 49.7% with ABEmax in the assay (Fig. 2c, d; Additional file 5: Table S4). We then merged the editing outcome at amino acid level, i.e., all the edited reads leading to the same amino acid change were counted as the same outcome (Fig. 2a). After merging, the fraction of sgRNAs associated with only one or two outcomes increased from 60.2 to 88.8% for AncBE4max and from 64.4 to 83.2% for ABEmax (Fig. 2c,d; Additional file 5: Table S4). We also found that more than 75% sgRNAs had a major outcome that was supported by over 80% of the editing products (Fig. 2e). Therefore, we defined the editing result with the highest frequency as the variant associated with an sgRNA (Additional file 6: Table S5). For each sgRNA, we then calculated the editing efficiency as the fraction of reads with the variant we defined. We observed good correlation between biological replicates for the editing efficiencies of the sgRNAs (Additional file 1: Figure S3e, f, Pearson’s *r* = 0.946 for AncBE4max and *r* = 0.886 for ABEmax).

To demonstrate that the strategy of introducing the exogenous vectors in HEK293T could reveal the endogenous editing outcomes, we used a targeted amplicon sequencing assay to directly measure the editing outcomes and efficiencies in the genomes of eHAP cells. We found a statistically significant correlation between the endogenous editing efficiencies and the ones on the integrated target sequences (Fig. 2f, g, Pearson’s *r* = 0.445 and 0.531 for AncBE4max and ABEmax, respectively). The consistency with endogenous editing efficiencies may be improved by performing the integrated target site assay in eHAP cells. The editing efficiencies were also in agreement with the predicted editing activities of sgRNAs using the recently published editing outcome and efficiency predictor BE-Hive [25] (Additional file 1: Figure S4a, b, Pearson’s *r* = 0.455 and 0.454 for AncBE4max and ABEmax, respectively).

### Modeling functional scores with sgRNA editing activity correction

**Fig. 3:**
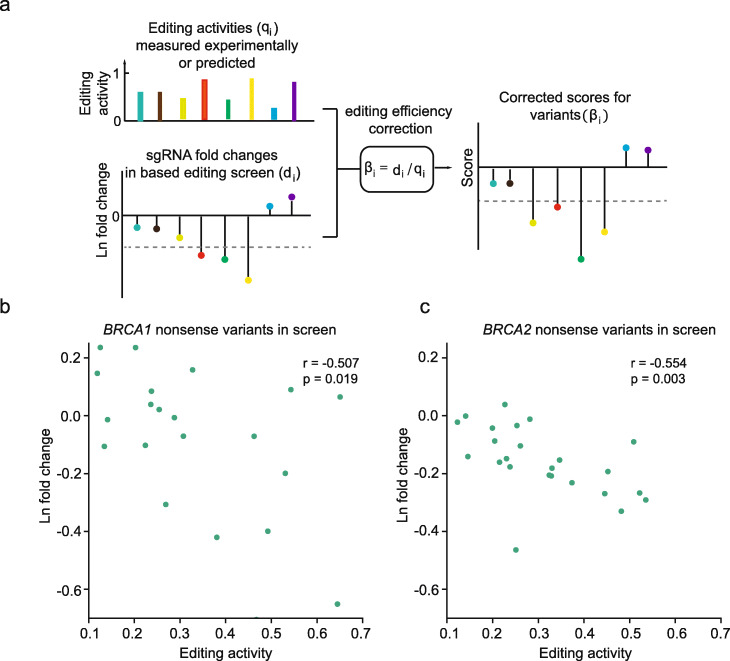
Modeling the dependence of sgRNA fold changes on sgRNA activity in base editing screens. **a** Schematic of the efficiency correction model for calculating the functional scores. **b**, **c** Experimental validation of the negatively correlated relationship between ln fold change and sgRNA editing activity (the relative editing efficiency for generating the variant), using a group of sgRNAs that could generate nonsense mutations in *BRCA1* (**b**) or *BRCA2* (**c**). Pearson’s correlation coefficients are shown

To precisely determine the functional effects of variants, we proposed a model to correct the effects of sgRNA editing activity in our base editing screen (Fig. 3a). For each variant that is the most frequent product after a sgRNA editing, we assumed that the observed depletion of the sgRNA is linearly correlated with the editing efficiency for generating the variant. Specifically, for each sgRNA *i* in the screen experiment *j*, the functional score for the most frequent variant generated by sgRNA *i* (variant *i*) in experiment *j*, *β*_*ij*_, can be modeled as:

$$ {\beta}_{ij}={d}_{ij}/{q}_i $$

here *d*_*ij*_ is the ln fold change of sgRNA *i* in experiment *j*, relative to the median ln fold change of negative control sgRNAs in that experiment. *q*_*i*_ is the relative editing activity for sgRNA *i* to generate variant *i*, which can be measured experimentally or predicted computationally. We also showed that the linear relationship could be approximated from a simple exponential cell growth model at certain conditions (See “Methods,” “Modeling functional scores of variants with editing efficiency”).

Consistent with the model, for the sgRNAs that could generate premature stop codons in *BRCA1*, which may be equivalent to a group of nonsense variants that having the same *β* score, we observed an inverse linear relationship between ln fold change and the relative efficiency of nonsense variant production (Fig. 3b, Pearson’s *r* = − 0.507, *p* = 0.019). Similar trend was also observed for sgRNAs that could generate nonsense variants in *BRCA2* (Fig. 3c, Pearson’s *r* = − 0.554, *p* = 0.003).

**Fig. 4:**
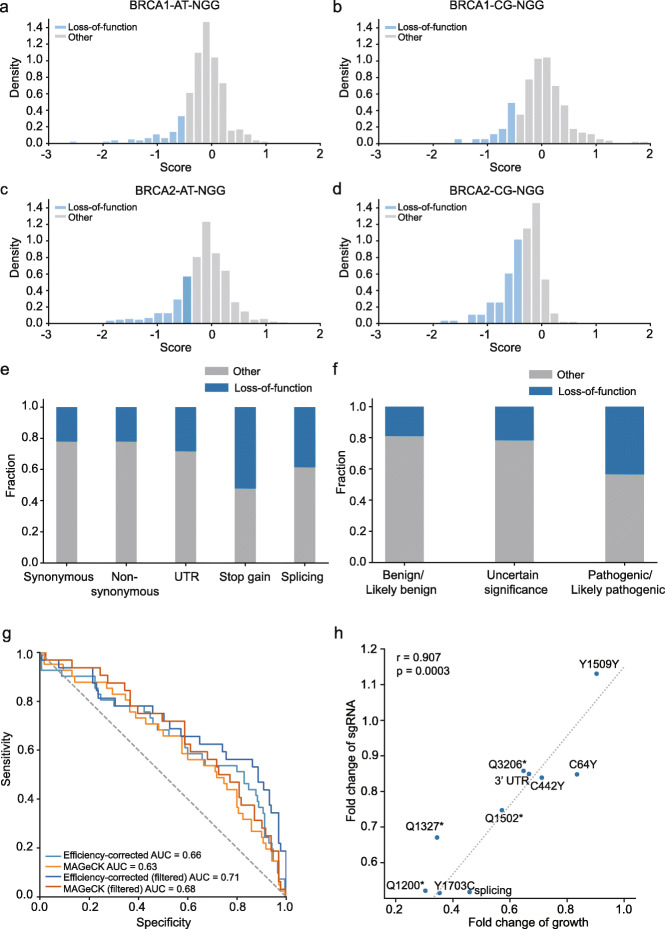
Classification of loss-of-function variants using efficiency-corrected scores. **a–d** Histograms of score distributions for variants tested in four screens: BRCA1-AT-NGG (**a**), BRCA1-CG-NGG (**b**), BRCA2-AT-NGG (**c**), BRCA2-CG-NGG (**d**). Blue bars represent variants that are classified as loss-of-function variants using thresholds determined by Gaussian mixture modeling. **e** Ratio of loss-of-function variants in different mutational categories. **f** Ratio of loss-of-function variants within different *ClinVar* interpretation categories. **g** ROC curves for loss-of-function variant classification using our efficiency correction model and MAGeCK. The orange and light blue lines used 49 “pathogenic/likely pathogenic” variants in *ClinVar* as true positives and 190 “benign/likely benign” variants as true negatives. The red and blue lines used 37 “pathogenic/likely pathogenic” variants and 146 “benign/likely benign” variants, after filtering the sgRNAs that had editing activity lower than 0.2. **h** Fold change of sgRNAs for 7 loss-of-function variants in the screens, as well as the fold change of three control variants in Fig. 1b, is plotted against the fold change of cell growth in the cell growth assays. Pearson’s correlation coefficient is shown. Detailed data for the figure is presented in Table S1

Using the efficiency correction model, for each sgRNA, we calculated the functional score for the major outcome generated by that sgRNA (Fig. 3a; Additional file 7: Table S6). To identify loss-of-function variants, we chose the mixture model of four Gaussian components to fit the score distributions for each screen (Additional file 13: Supplementary Note 1). For each library, the majority of scores followed a normal distribution with a mean at about zero, consistent with our assumption that majority of variants have no impact on *BRCA1/2* function. The two subpopulations following distributions with negative means were likely corresponding to the variants that having deleterious effects on gene function (Additional file 1: Figure S5a-d). We classified the variants as “loss-of-function” if their probability belonging to any of the two negative-mean distributions were more than 0.8 (See “Methods,” “Identification of loss-of-function variants using Gaussian mixture model” and Additional file 13: Supplementary Note 1). The equivalent *β* score cutoffs were − 0.55, − 0.45, − 0.37, and − 0.4 for *BRCA1_AT_NGG*, *BRCA1_CG_NGG*, *BRCA2_AT_NGG.* and *BRCA2_CG_NGG* libraries, respectively. In total, 121 and 290 variants were identified as loss-of-function mutations for *BRCA1* and *BRCA2*, respectively (Fig. 4a–d). We estimated that the FDR for LOF variants was at a level of 0.2 (Additional file 13: Supplementary Note 1).

As expected, the fraction of loss-of-function mutations in *BRCA1/2* was higher in the “stop gain” group comparing to other groups (52.2% compared to 23.1% in other groups; Fisher’s exact test, *p* = 4.9 × 10^− 6^) (Fig. 4e). Enrichment of deleterious mutations was also observed in the “splicing” group, which referred to variants within 2 bp of the exon-intron junction (Fig. 4e). Comparing to the classifications in the *ClinVar* database, 46.9% of the 49 “pathogenic/likely pathogenic” variants were classified as “loss-of-function” in our screen [4] (Fig. 4f). The proportions of loss-of-function variants were lower in the “benign/likely benign” and “variants of uncertain significance” groups, counted as 18.3% and 22.8%, respectively. Our loss-of-function variants overlapped significantly with previously reports (Additional file 8: Table S7, Fisher’s exact test, *p* < 0.005). Ten of our *BRCA1* LOF variants were also classified as deleterious variants in a saturated genome editing [11]. And we identified 8 of the 17 LOF variants in a base editing screen targeting *BRCA1* [21].

To evaluate the performance of our efficiency-corrected model on distinguishing true loss-of-function variants from other variants, we calculated the sensitivity and specificity of our classification using “pathogenic/likely pathogenic” variants in the *ClinVar* database as true positives and “benign/likely benign” variants in *ClinVar* as true negatives. Compared to MAGeCK, the area under the receiver operator characteristic curve (ROC-AUC) was higher in our method (0.66 vs. 0.63, Fig. 4g). At a specificity ranging from 0.8 to 0.9, our sensitivity is about 1.5- to 1.8-fold higher than MAGeCK (Fig. 4g). These results demonstrated our efficiency correction strategy improved the accuracy for identification of loss-of-function variants. Filtering out the sgRNAs that had lower editing activity further increased the sensitivity from 46.9 to 51.4% in our model (Fig. 4g), suggesting that increasing editing efficiency of base editing may improve the sensitivity of the screen.

To validate the newly identified loss-of-function variants, we performed cell viability assays on 10 randomly selected LOF variants, including three nonsense variants (Additional file 2: Table S1, Fig. 4h). For 7 of the 10 LOF sites, treating cells with sgRNAs resulted in the delayed growth of eHAP cells compared with non-targeting controls (Additional file 2: Table S1, Fig. 4h). And the level of delayed growth correlated with the degree of sgRNA deletion in the screen (Fig. 4h, Pearson’s correlation coefficient *r* = 0.907, *p* = 0.0003).

### Functional assessment of variants in *BRCA1/2* using NG base editors

**Fig. 5:**
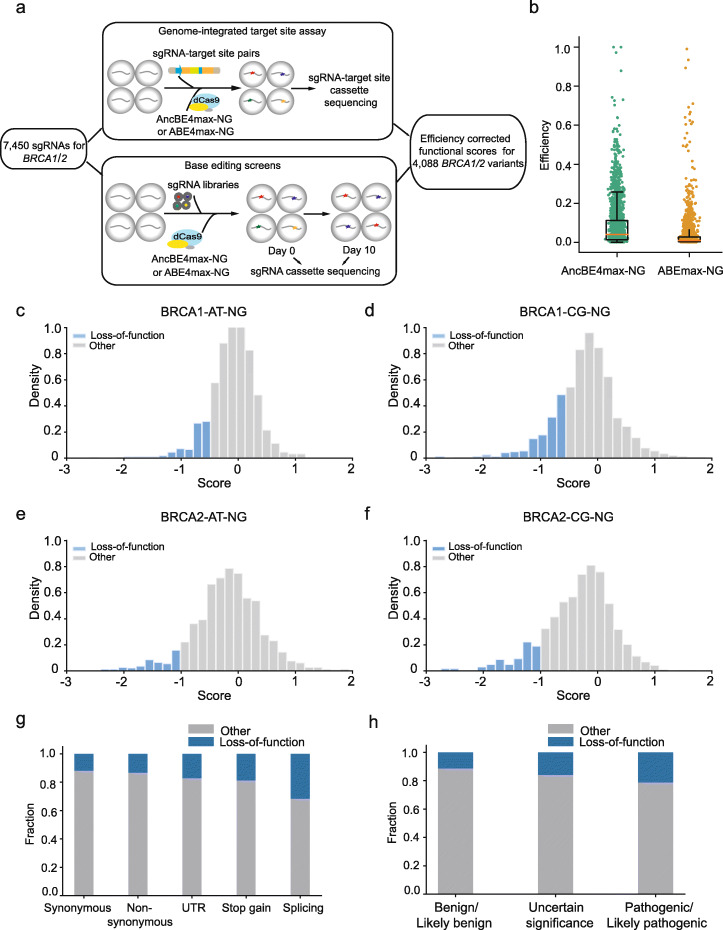
Functional screens of 4088 variants in BRCA1/2 using base editors recognizing “NGN” PAM sequence. **a** Schematic overview of the base editing screens with “NGN” base editors and efficiency correction. **b** Editing efficiencies for AncBE4max-NG and ABEmax-NG base editors measured by the genome-integrated target site library assay. The orange line represents median of the fraction of edited reads for all sgRNAs in the library. **c–f** Histograms of score distributions for variants tested in four screens: BRCA1-AT-NGG (**c**), BRCA1-CG-NGG (**d**), BRCA2-AT-NGG (**e**), BRCA2-CG-NGG (**f**). **g** Fraction of loss-of-function variants among all the tested variants in different mutational categories

Having fully established the assay, we next applied the method to assess 7450 bases in *BRCA1/2*. We modified seven amino acids in the AncBE4max and ABEmax to generate base editors AncBE4max-NG and ABEmax_NG that recognizing “NGN” as the PAM sequence for the screen [18]. We performed the base editing screen experiments in four separate pools and measured the editing activities of sgRNAs with genome-integrated target site library assay (Fig. 5a; Additional files 3 and 6: Table S2, S5). In general, “NGN” recognizing base editors AncBE4max-NG and ABEmax_NG had lower efficiencies than “NGG” recognizing base editors AncBE4max and ABEmax, with the median fraction of edited reads ranging from 1.0 to 3.8% (Fig. 5b; Additional file 1: Figure S6a,b). Therefore, sgRNAs with low editing activities may not be able to generate a measurable fold change in this system. After filtering for the sgRNAs that have low editing activities (scaled activity less than 0.2), we associated 567 variants with negative effects on the function of *BRCA1/2* (Fig. 5c–f), the estimated FDR for these variants were under 0.1 (Additional file 13: Supplementary Note 1). Among the 713 variants that were shared between screens using “NGG” recognizing base editors and screens using “NGN” recognizing base editors, about 74% of them were concordant in functional classification, including 32 loss-of-function variants (Additional file 9: Table S8, Fisher’s exact test, *p* = 0.004). As expected, deleterious mutations were enriched in the “stop gain” and “splicing” group, compared to the synonymous group (Fig. 5g). Among the 558 loss-of-function variants, 14 of them were reported as “pathogenic/likely pathogenic” in the *ClinVar* database, and 101 of them were labeled as VUS or “benign/likely benign” (Fig. 5h). The sensitivity for identifying “pathogenic/likely pathogenic” variants was 2.2-fold lower for screens with NG base editors compared to screens with NGG base editors, likely due to the low efficiency of NG base editors.

### Deleterious variants reveal different mechanisms for functional loss of *BRCA1/2*

**Fig. 6:**
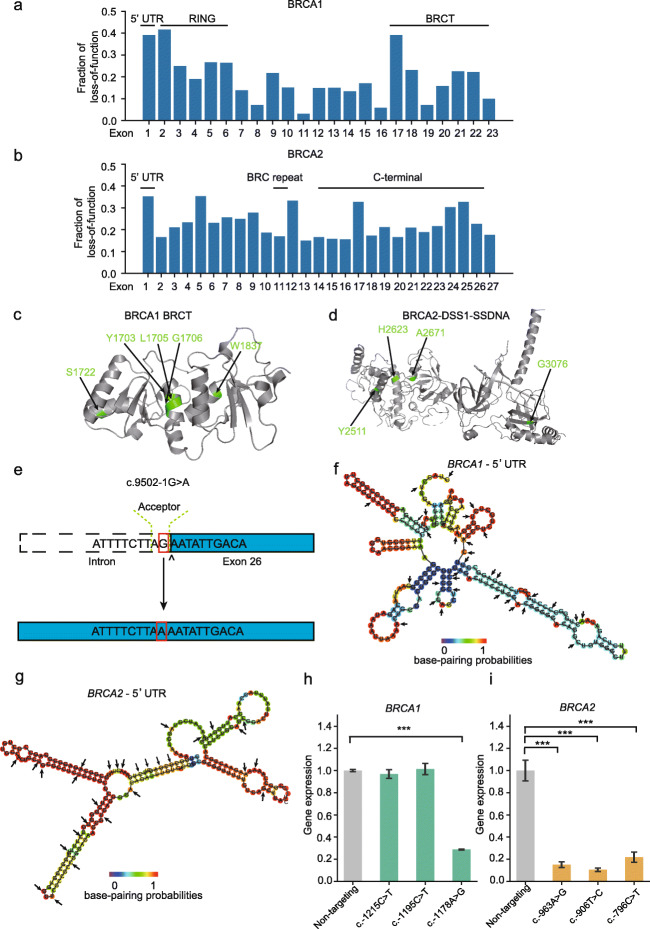
Identified loss-of-function variants were in both coding and noncoding regions of *BRCA1/2* genes. **a**, **b** Fraction of identified loss-of-function variants among all the tested variants across the exons of *BRCA1* (**a**) or *BRCA2* (**b**). **c** Identified loss-of-function variants in the BRCT domains of *BRCA1* (PDB code: 1 T29). Variant positions are shown in green. **d** Four loss-of-function variants viewed on the mouse BRCA2-DSS1-ssDNA (PDB code: 1MJE). Residues corresponding to positions of five variants in human are shown in green. **e** Schematic of the c.9502-1G>A variant disrupting the splicing acceptor site in exon 26. **f**, **g** Secondary RNA structures predicted by RNAfold for the 232 nucleotide in the 5′ UTR of *BRCA1* (**f**) and the 228 nucleotides in the 5′ UTR region of *BRCA2* (**g**). The structure is colored by base-pairing probabilities. For unpaired regions the color represents the probability of being unpaired. Variants identified as “loss-of-function” in our screen were indicated by arrows. **h**, **i** Gene expression analysis after base editing for 6 sites in 5′ UTR regions of *BRCA1* (**h**) or *BRCA2* (**i**). Expression levels of *BRCA1* or *BRCA2* in treated group were normalized to non-targeting control groups. Error bars represent SEMs from 3 independent quantitative PCR experiments and two-tailed Student’s *t* tests were used to determine *P* values. Detailed data for the figure is shown in Table S1

Altogether, excluding nonsense mutations, 343 *BRCA1* variants and 530 *BRCA2* variants were identified as loss-of-function variants through our base editing screens. The identified deleterious variants were distributed unevenly across the exons (Fig. 6a,b). For *BRCA1*, the fraction of deleterious variants among all the tested variants was as high as 0.4 in exon 17, which encodes part of the conserved BRCT domain. A group of variants were clustered in residues constituting the BRCT–BRCT interface, such as Y1703C, L1705P, and G1706K (Fig. 6c). And mutations such as S1722P and W1837R may disrupt WXXXS motif in helix α3 domain in the BRCT domain [31] (Fig. 6c). The clustering of deleterious variants in known functional domain was also observed for *BRCA2*. For example, a cluster of them were in the DNA-binding domain (DBD) of *BRCA2*, which is responsible for binding both single-stranded and double-stranded DNA [3]. In structure of the BRCA2 DBD domain, H2623 (mouse H2544) and Y2511 (mouse Y2432) are residues of the helical domain, A2671 (mouse A2592) is a residue in the OB1 fold, G3076 (mouse G2995) is located on the OB2-OB3 interface. These four residues are well conserved in more than 5 orthologs [32], and mutations at these sites may disrupt the interaction of *BRCA2* and DNA (Fig. 6d). For example, G3076E has been confirmed to be a deleterious mutation that could change the homologous recombination function in HDR assay [33].

Other loss-of-function variants include the mutations within the canonical splicing sites that may affect *BRCA1/2* function at mRNA level. As shown in Fig. 6e, BRCA2_G_32971034_NGG induced a G to A mutation that may destroy the acceptor splice site in exon 26 for *BRCA2* (Fig. 6e). These variants may cause aberrant splicing of the genes.

We also identified many mutations in the untranslated regions of the gene. We found a high ratio of loss-of-function variants in the first exon of *BRCA1*, which encode the 5′ UTR region of the gene (Fig. 6a). Variants generated by 32 of the 86 sgRNAs targeting the 232 bp 5′ UTR region were classified as loss-of-function for *BRCA1*. Similarly, 29 of the 87 sgRNAs targeting the 228 bp 5′ UTR region in *BRCA2* were identified to induce functional loss after base editing. Many strong base pairings were predicted by RNAfold [34] in both 5′ UTRs, which may be responsible for forming secondary RNA structures for translation regulation and mRNA stability [35] (Fig. 6f,g). For 6 sgRNAs that inhibited the growth of eHAP cells in a cell viability assay (Additional file 1: Figure S7), we found that four of them reduced the mRNA levels of *BRCA1* or *BRCA2* (Fig. 6h), suggesting that their associated variants had direct impacts on *BRCA1/2* function through regulation at the mRNA level. One of the four sgRNAs is associated with a single nucleotide variant in *BRCA2* (c.-796C>T), which has been previously reported in patients with familiar breast and ovarian cancer, yet were classified as VUS in *ClinVar* (Additional file 7: Table S6). Our results suggested that the variant may need to be re-evaluated for their clinical significance.

## Discussion

Identification of pathogenic nucleotide variants in cancer genes have direct impact on medical management decisions, including cancer risk assessment and personalized treatment. Here we demonstrated the feasibility of base editing screens in assessing pathogenicity of variants by assaying 4861 mutations in *BRCA1* and *BRCA2* genes. We were able to identify 31 known pathogenic mutations as well as 377 unreported missense mutations that could negatively impact *BRCA1/2* function. These deleterious mutations distributed in both coding and noncoding regions of *BRCA1/2*.

Due to the large size of *BRCA1/2* genes, previous functional assays for variants were limited to SNVs in a few known functional domains, such as the RING and BRCT domains of *BRCA1* [11]. Here we showed that base editing screens could systematically identify functional variants in all regions by assaying mutations in their native genomic context. For example, we identified a group of variants clustered in the 5′ UTR of *BRCA1/2*. The 5′ UTR region contains key elements for multiple levels of post-transcriptional regulation [36]. The LOF variants in the 5′ UTR regions may compromise *BRCA1/*2 function through different mechanisms. We identified a cluster of *BRCA1* LOF variants located in a predicted internal ribosome entry site (IRES), which is a regulatory secondary structure that could initiate cap-independent translation. We also found that several variants in the 5′ UTR regions resulted in reduced mRNA levels of *BRCA1/*2. A recent study showed that some variations in the 5′ UTR sequences increased mRNA decay using a high-throughput synthetic reporter assay [35]. It is possible that these variants we found also altered the stability of *BRCA1/*2 mRNAs. With further investigations on these variants, our findings may impact future classification of variants in 5′ UTRs during genetic testing.

One obstacle in applying high-throughput base editing screens for functional mapping of variants was the complex outcomes and variable editing efficiencies of sgRNAs. Here we addressed the problem by experimentally determining the editing outcome and efficiency and incorporated them into a model for calculating functional scores. We have shown that outcome mapping has allowed the association of sgRNA effects with the correct mutations. We have also shown that the sgRNA depletion readout inversely correlated with sgRNA activity and a model with editing efficiency correction could significantly improve the quantitative scoring of variant effects. With outcome mapping and efficiency correction, we were able to achieve an over 1.5-fold increase in sensitivity for identification of loss-of-function variants at a specificity of 80%.

Our efficiency-correction framework is readily applied to identification of LOF variants in other cancer genes. Genes that are essential in eHAP cells include cancer risk genes such as *PALB2*, *BARD1*, *RAD51C*, and *RAD51D*, which have thousands of variants recorded in *ClinVar* [4]. About two-thirds of the variants in these genes could be directly assessed using the same approach presented in this study. For genes that are not essential in eHAP cells, base editing screens with the efficiency correction is still applicable if a proper functional assay is developed to enrich or deplete the LOF variants. To simplify the workflow, in the future, instead of using empirically determined editing efficiencies, the in silico-predicted sgRNA activities may be used for editing efficiency correction. It has been shown that the relative activity of sgRNA on editing was mostly dependent on the sequence of the sgRNA and the interplay with base editors [25], and machine learning models have been developed to predict the editing activities and outcomes [25, 37]. It is noted that although efficiency correction is needed for better identification of loss-of-function variants in all BE screen experiments, the linear relationship between ln fold change and editing efficiency may not directly apply for other base editing screens. Other efficiency correction models may be developed under our framework to improve variant classification.

Although our approach has addressed the problem of variability in guide RNA editing efficiency, several problems with current versions of base editors set the limitations for functional assessment of variants using BE screens. First, the targeting scopes of base editors are constrained. Only four types of transition point mutations are available for current CBE or ABE base editors. And current versions of base editors based on Cas proteins still require a PAM sequence, which further limits the number of sites that could be edited [38]. Second, base editors can modify more than one nucleotide in the editing activity window, preventing the assessment of some single nucleotide variants. Further, only one sgRNA was used for each variant, which could lead to low sensitivity for identifying LOF variants. For the CRISPRi screens, when only one sgRNA was used, it has been estimated that the sensitivity for identifying essential genes to be less than 0.2 [39]. Moreover, the low editing efficiency for some of the base editors reduced the sensitivity for identifying known pathogenic variants, as we have observed for the “NGN” recognizing base editors in comparison to “NGG” recognizing base editors. In addition, the off-target effects of base editors are also a potential concern [40–42]. Therefore, base editors with broader targeting scope, more precision, and higher editing efficiencies are needed. Recently developed “prime” editor, which uses a reverse transcriptase and a template RNA sequence in sgRNA to introduce mutations in the genome, are more precise and less restrained by a PAM sequence [43]. The prime editors can generate all 12 possible types of point mutations, making it possible for assaying genetic changes at any position of the genome. These developments could greatly facilitate the high-throughput analysis of variants, which could benefit interpretation of variants in cancer samples for better risk assessment and therapeutic applications.

## Conclusions

In summary, we developed a framework to combine base editing screens with sgRNA efficiency mapping for better functional assessment of variants. Our results demonstrated the potential of base editing screens for functional mapping of variants, especially for noncoding variants assayed in their native genomic context. The application of the method to other cancer genes will help the clinical evaluation of variants in cancer.

## Methods

### Plasmid construction

PCR primers used in this study are listed in the Additional file 10: Table S9. PCRs were performed using 2 X TransStart FastPfu PCR SuperMix (TransGen). The digestion enzymes were all purchased from Thermo Fisher Scientific. Plasmids were constructed by Gibson Assembly (NEB) unless specified. The plasmid sequences were all confirmed by Sanger sequencing.

The four base editor constructs were generated with DNA fragments amplified from three vectors: pCMV_AncBE4max (Addgene 112094), pCMV_ABEmax (Addgene 112095), and pLenti-BE3RA-P2A-GFP-PGK-Puro (Addgene 110,868). Specifically, mutations in the SpCas9 protein were introduced using overlap extension PCR to generate NG_SpCas9 base editor fragments AncBE4max_NG and ABEmax_NG. AncBE4max_NG was then inserted with EcoRV-digested pCMV_AncBE4max to generate pCMV_AncBE4max_NG. ABEmax_NG was inserted into pCMV_ ABEmax at EcoRI and EcoRV sites to generate pCMV_ ABEmax_NG. In order to introduce a selection marker in the base editor constructs, P2A_EGFP were PCR amplified from pLenti-BE3RA-P2A-GFP-PGK-Puro and assembled with BshTI-linearized pCMV_AncBE4max, pCMV_ABEmax, pCMV_AncBE4max_NG, or pCMV_ABEmax_NG to generate AncBE4max_P2A_EGFP, ABEmax_P2A_EGFP, AncBE4max_NG_P2A_EGFP, and ABEmax_NG_P2A_EGFP respectively.

All CRISPR gRNAs used in this project were cloned into lentiGuide-Puro (Addgene 52963) as described previously [44].

To generate the constructs for measuring editing efficiency using the integrated-genome target site assay, lentiGuide-Puro was digested by restriction enzymes Esp3I and SmaI. An insert segment

(ATCTTGTGGAAAGGACGAAACACCGGAGACGGTTGTAAAGCTTGGCGTAACTAGATCTTGAGACAAATGGCAGTATTCATCCACAATTTTAAAAGAAAAGGGGGGATTGGGGGGTACAGTGCAGGGGAAAGAATAGTAGACATAATAGCAACAGACATACAAACTAAAGAATTACAAAAACAAATTACAAAAATTCAAAATTTTCGGGTTTATTACAGGGACAGCAGAGATCCACTTTGGCGCCGGCTCGAGGGGGCCCGGGTGCAAAGATGGATAAAGT) was synthesized using GenPart DNA Synthesis service from the GenScript company. The segment was then assembled with the linearized lentiGuide-puro vector.

### Oligonucleotide library design and synthesis

The exon sequences of *BRCA1* and *BRCA2* were downloaded from NCBI genebank (hg 19) and searched for targetable sites (C or A) within the 13–17-bp window upstream of an NGG or NG PAM sequence using a custom python script. If multiple sgRNAs were available for one site, the one farthest to the 5′ of the PAM sequence was selected. In total, 4034 sgRNAs targeting *BRCA1* and 5324 sgRNAs targeting *BRCA2* were designed. The off-target sites for each sgRNA were predicted using Cas-OFFinder [45], without mismatch in protospacer.

Two oligonucleotide libraries containing 4946 and 6149 oligos were designed for the base editing screen. sgRNAs were divided into 8 subpools according to their targeting genes and the base editor type for their target sites. A 20-bp barcode sequence designating the subpool information were arranged at both the 5′ and 3′ end of each sgRNA sequence. Each oligonucleotide also consisted of two 20-bp primer sequences at either end of the barcoded sgRNA sequence for PCR amplification. Five percent and 10% non-targeting sgRNAs were included as negative control sgRNAs for NG and NGG pools respectively. In total, 230 sgRNAs that may generate new stop codons in known essential genes were included in the four pools targeting C bases as additional controls for the screen.

An oligonucleotide library with 7589 sequences were designed for evaluating the sgRNA activities using the integrated-genome target site assay. For this pool, each sgRNA in the base editing screen library was paired with its target sequence and arranged in one oligonucleotide with the following elements: a 20-bp sgRNA sequence, an 82-bp sgRNA scaffold, and a 26-bp targeting sequence with a PAM (NGN or NGG) and 3 bp upstream of protospacer.

The three oligo pools were synthesized on 12 K arrays and purchased from GenScript company. All the sgRNA sequences and their corresponding oligos are listed in Supplementary information (Additional files 3 and 4: Table S2, S3).

### Library construction

The eight libraries for performing the base editing screen was amplified following a two-step PCR protocol using NEBNext High-Fidelity 2X PCR Master Mix (NEB). The first PCR step was performed with specific primers for each subpool to amplify oligo sequences, using 1 ng template for 18 cycles. In the second PCR amplification step, extension primers were used to remove specific primer regions and amplify protospacer with assembly homologous arms, using 2 μl first step PCR products for 12 cycles. PCR products were purified with Zymoclean Gel DNA Recovery kit (ZYMO) for the following assembly reactions. The lentiGuide-puro vector was first linearized via Esp3I (Thermo Scientific), then assembled with purified second step PCR products using NEBuilder HiFi DNA assembly master mix (NEB).

To generate the library for evaluating the sgRNA activities, 50 ng synthesized oligos (GenScript) were PCR amplified for 25 cycles using NEBNext High-Fidelity 2× PCR Master Mix (NEB). The library PCR products were purified using Zymoclean Gel DNA Recovery kit (ZYMO) and assembled with the editing efficiency constructs linearized by Esp3I using NEBuilder HiFi DNA assembly master mix (NEB).

The Gibson Assembly products were purified with DNA Clean & Concentrator-5 kit (ZYMO) and electroporated into MegaX DH10B T1 Electrocomp competent cells (Thermo Scientific). After growing at 32 °C for 14 h, the colonies were scraped for subsequent plasmid preparation using ZymoPURE II Plasmid Midiprep Kit (ZYMO) according to the manufacturer’s instructions.

### Cell culture

HEK293T cells were cultured in Dulbecco’s modified of Eagle’s medium (Corning) supplemented with 10% fetal bovine serum (Thermo Fisher Scientific) and 1% penicillin-streptomycin (Thermo Fisher Scientific), and cells were grown at 37 °C with 5% CO_2_. Human eHAP cells (Horizon discovery, #C669) were purchased and maintained in Iscove’s modified Dulbecco’s medium (IMDM) with l-glutamine and 25 mM HEPES (Thermo Fisher Scientific), also supplemented with 10% fetal bovine serum and 1% penicillin-streptomycin. eHAP cells are haploid cells that may revert to diploid states during cell culture. To sort 1 N-enriched eHAP cell population, cells were stained with Hoechst 33342 (Merck) at a concentration of 5 μg/ml at 37 °C for 20 min. Fluorescent-activated cell sorting (FACS) was performed to sort 1 N ploidy according to the lowest Hoechst peak using a MA900 cell sorter (Sony Biotechnology).

### Lentivirus production

HEK293T cells were grown on 150-mm dishes (CORNING) coated with poly-l-lysine (Sigma) until 90% confluency. For each 150-mm dish, 21 μg of the library plasmids, and 15 μg of psPAX2 (Addgene 12,260) and 6 μg of pMD2.G (Addgene 12,259) were transfected into HEK293T cells using 63 μl of Neofect DNA transfection reagent (Neo Biotech). At 16 h post transfection, the culture medium was changed with viral production medium (Lonza). Virus supernatant was collected 40 h post transfection, filtered with a 0.45-μm Sterile Filter Unit with Durapore PVDF Membrane (Millipore), aliquoted, and stored at − 80 °C before use.

### Base editing screens

For every library, 12 million eHAP cells were transduced at an MOI of 0.3 in a 150-mm dish. After 48 h, cells were split into four dishes with puromycin-supplemented medium (1 μg/ml) for 48 h. Following antibiotic selection, eHAP cells were allowed to recover for 24 h. One tenth of the cells were collected to use as cells at T0, and the remaining cells were placed in culture to proliferate 90% confluency. The cells were transfected with 40 μg base editing vectors using 160 μl FuGENE HD Transfection Reagent (Promega). After 48 h, cells were sorted on a SH800S cell sorter (Sony Biotechnology) to enrich cells with enhanced EGFP expression. After 10 days, cells were harvested for genomic DNA sequencing.

### Genomic DNA sequencing for sgRNAs in base editing screen

Cells in − 80 °C were thawed and genomic DNA were extracted using DNeasy Blood and Tissue kit (Qiagen). Sequences of sgRNAs were amplified by a two-step PCR reaction. For each sample, in the first PCR, four separate reactions were performed with a total of 8 μg genomic DNA as the template. The following primer sequences were used to amplify sgRNAs:

- CriV2-firstround_F: AATGGACTATCATATGCTTACCGTAACTTGAAAGTATTTCG;
- CriV2-first round_R1:
- GTGACTGGAGTTCAGACGTGTGCTCTTCCGATCTACTGACGGGCACCGGAGCCAATTCC.

In the second PCR, two separate reactions were performed using 2 μl first reaction product as the template. The following primers were used to add sequencing adaptor and index sequences:

- Cri_library_F: AATGATACGGCGACCACCGAGATCTACACTCTTTCCCTACACGACGCTCTTCCGATCTTCTTGTGGAAAGGACGAAACACCG;
- HiSeq_Index: CAAGCAGAAGACGGCATACGAGATNNNNNNGTGACTGGAGTTCAGACGTG (NNNNNN represents a 6 bp index).

Amplification was performed with 20 cycles for the first step PCR and 10 cycles for the second PCR using NEBNext High-Fidelity 2× PCR Master Mix (NEB). The PCR products were purified with Zymoclean Gel DNA Recovery Kit (ZYMO) and quantified by Qubit dsDNA High Sensitivity kit (Life Technologies). Sequencing was performed on an Illumina Hiseq X Ten.

### Integrated-genome target site library assay for outcome and efficiency mapping

HEK293T cells were transduced with lentivirus from the sgRNA activity library at an MOI of 0.5. At 48 h post-infection, cells were selected with 1.5 μg/ml puromycin for 2 days to enrich the transduced cells, which were then transfected with the four base editors, AncBE4max_P2A_EGFP, ABEmax_P2A_EGFP, AncBE4max_NG_P2A_EGFP, or ABEmax_NG_P2A_EGFP, respectively. EGFP-positive cells were collected using FACS and the genomic DNA (gDNA) was isolated using the Blood & Cell Culture DNA Midi Kit (Qiagen). To identify the sgRNA inserts and the matched integrated-genome target sites, we performed 22 separate PCR reactions (3 μg gDNA/ 50 μl reaction volume) for each sample with NEBNext High-Fidelity 2× PCR Master Mix (NEB) and combined the amplicons. The amplicons were gel purified with Zymoclean Gel DNA Recovery Kit (ZYMO) and sequenced on a Hiseq Xten platform. Primers containing both Illumina adaptor and barcode sequences are listed in Additional file 10: Table S9.

### Targeted amplicon sequencing for endogenous editing efficiency mapping

eHAP cells were placed in 10-cm dishes to proliferate to 80% confluency and then transfected with the 10 μg sgRNA libraries and 20 μg base editors using 120 μl FuGENE HD Transfection Reagent. After 48 h, cells were sorted on Sony SH800S to enrich cells with EGFP expression. Genomic DNA was extracted and amplified using a custom panel targeting all exons in *BRCA1/2* (Genetronhealth), and sequenced on X Ten with a median average depth of 50,000. After removing low-quality bases and sequencing adapters by Trimmomatic (0.33) [46] with parameters: “TRAILING:3 SLIDINGWINDOW:4:15 MINLEN:36,” clean sequencing reads were aligned to reference sequences consisting of the full length of *BRCA1* and *BRCA2* genes (hg19), by using BWA mem (0.7.10) [47]. Variant detection was performed according to the pileup format file generated by Samtools mpileup (1.3.1). The endogenous editing frequency was calculated using the same formula as that for “fk” in “Editing outcome and efficiency mapping using data from integrated-genome target library assay.” Sites with significantly higher mutation frequency in the edited samples than in the control samples were retained.

### *BRCA 1/2* essentiality test with RNAi in eHAP cells

Sequences of two *BRCA1* targeting siRNAs and two *BRCA2* targeting siRNAs are as follows: *BRCA1–2*: CCACACGATTTGACGGAAA, *BRCA1–3*: CTACTCATGTTGTTATGAA. BRCA2–1: TAAATTTGGACATAAGGAGTCCTCC, and BRCA2–2: GAAGAACAATATCCTACTA. On day 1, 5 × 10^5^ eHAP cells were plated on 6-well dishes until 60% confluency, and 3 μl siRNAs (20 μM) were transfected into cells using Liopofectamine RNAimax Reagent (Invitrogen).

Twenty-four hours after transfection, 1000 cells were plated on a 96-well plate, and the remaining cells were harvested to extract total RNA. First-strand cDNA synthesis was performed with SuperScript® III First-Strand Synthesis SuperMix (Thermo Fisher Scientific) with 2 μg total RNA as template. Quantitative Real-time PCR was performed on RocheLightCycler480 with 1 μl reverse transcription products. GAPDH was used as the internal control. The primer sequences used were as follows:

- BRCA1-qPCR_F: GTCCCATCTGTCTGGAGTTGA;
- BRCA1-qPCR_R: AAAGGACACTGTGAAGGCCC.
- BRCA2-qPCR_F: AAGCACTCCAGATGGCACAAT;
- BRCA2-qPCR_R: TCTTGACCAGGTGCGGTAAAA.

For cell viability assay, 1000 eHAP cells were plated into 96-well plates and then cultured for 4 days at 37 °C with 5% CO_2_. Each well was added 100 μl medium of CCK-8 solution (Dojindo) and IMDM, mixed at 1:10 ratio. The absorbance value was measured in a microplate reader FlexStation 3 (Molecular Devices) at 450 nm.

### Cell viability assays for validating sgRNA functional effects

eHAP cells were infected with lentivirus containing sgRNAs targeting *BRCA1/2* and selected by puromycin for 2 days. For each sgRNA functional validation experiment, 4 × 10^6^ eHAP cells were transfected with the Neon Electroporation System for four times. For each electroporation, a mixture containing 1 × 10^6^ eHAP cells resuspended in Buffer R and 4 μg base editor expressing vectors were electroporated at 1350 V with 4 pulses of 10 ms. All the electroporated cells were combined and plated in a 10-cm dish. Twenty-four hours after electroporation, cells were sorted by Sony SH800S to enrich EGFP expressing population and cultured for another 24 h. Then 1000 cells were plated into 96-well plates for CCK-8 assay. Each assay was performed in triplicate and each experiment was repeated 3 times. For Sanger sequencing, targeting site flanking sequence was amplified, and the substitution frequency was analyzed by BEAT [48].

### Base editing screen fold change analysis

The sequencing reads were mapped and the read count for each sgRNA was calculated by MAGeCK (0.5.9.2) [49]. The sgRNA counts were then normalized through a median-of-ratio method using a normalization constant *w*_*j*_, which was estimated as the geometric mean of the read counts for all the negative control sgRNAs in the screen *j*. The fold change of sgRNA *i* was then calculated as normalized read count for sgRNA at day 10 (*C*_*ij*_) divided by the normalized read count at day 0 (*S*_*ij*_).

### Editing outcome and efficiency mapping using data from integrated-genome target library assay

Illumina sequencing raw reads R1 and R2 were first merged by SeqPrep with the following parameters: “-M 0.1 -m 0.001 -q 20 -o 20.” The reads were then aligned to the scaffold sequence (gttttagagctagaaatagcaagttaaaataaggctagtc-cgttatcaacttgaaaaagtggcaccgagtcggtgctttttt) using bwa mem (0.7.10) with the parameters -k 4 and -M. Reads containing the scaffold sequence were extracted using pysam package with the following criteria: the number of mismatch bases less than or equal to 3, mapping quality score is equal to 60, and the flag value is equal to 0. The sgRNA and its targeting sequence was extracted as the 20 bases upstream and downstream of the scaffold sequence, respectively. To eliminate reads with unmatched guide RNA and target sequence, the string similarity was calculated between the guide RNA and target sequence in each read. The reads with Jaro Distance less than 0.75 were considered as result of recombination during library preparation and were removed from the library data. The remaining sequences were clustered with the designed sgRNA library using CD-HIT (4.6.8) [50] at a threshold of 0.85. Each clustered read group was assigned to one designed guide RNA based on the number of mismatch bases and the consistency of PAM sequence using an in-house Python script. For each group, a read was counted as an edit event if a possible base transition was observed at any of the nucleotide in the editing window of the target sequence. The possible base transitions include AT to GC for ABE and CG to TA for CBE. For each possible editing result *k*, the fraction of *k* (*f*_*k*_) was calculated using the following formula:

$$ {f}_k=\mathrm{mean}\left\{\frac{R_{kj}-{D}_j{h}_c}{\ {D}_j-{D}_j{h}_c}\right\} $$

Here, *R*_*kj*_ is the number of reads with the editing result *k* in edited sample *j*, *D*_*j*_ is the total number of reads in sample *j*, and *h*_*c*_ is the background error frequency for editing result *k*. *h*_*c*_ is calculated as the mean fraction of reads with mutations as in editing result *k* in the control samples.

The editing results were annotated by ANNOVAR [51] using transcripts NM_007294.3 and NM_000059.3 for *BRCA1* and *BRCA2*, respectively. The editing results with the same amino acid changes were merged, and the editing outcome was defined as the editing result with the highest fraction of reads. sgRNAs having editing outcome with coverage less than 30 reads or frequency below 0.005 in both replicates of the genome-integrated target site assay were removed for further analysis. For the convenience of comparison, each sgRNA was assigned to a specific mutational type according to their annotation, including UTR, splicing, stop gain, nonsynonymous, and synonymous. If multiple amino acid changes were induced in one editing outcome, we selected the mutational type in the following order: stop gain > splicing > nonsynonymous > synonymous.

### Modeling functional scores of variants with editing efficiency

For *N* base editing screens, for each of the M sgRNAs in one screen, *C*_*ij*_, the normalized read count of sgRNA *i* at the end of experiment *j*, comes from two populations of cells, the edited cells with the most frequent variant generated by sgRNA *i* (denoted as variant *i*), and the cells without the variant *i*. Suppose the cell growth is exponential, *C*_*ij*_ can be described in the following formula;

1 $$ {C}_{ij}={S}_{ij}{p}_{ij}{e}^{\left({b}_j+{f}_{ij}\right)}+{S}_{ij}\left(1-{p}_{ij}\right){e}^{b_j} $$

Here *S*_*ij*_ refers to the sequencing depth normalized initial read count of sgRNA *i* in sample *j*, *p*_*ij*_ refers to the percentage of cells with the variant *i* in experiment *j*. *b*_*j*_ is the growth factor for experiment *j*, and *f*_*ij*_ refers to the functional impact of variant *i* have on cell growth in experiment *j*. From (1) we have:

$$ \ln \left({C}_{ij}/{S}_{ij}\right)={b}_j+\mathit{\ln}\left(1+{p}_{ij}\left({e}^{f_{ij}}-1\right)\right) $$

Here *b*_*j*_ can be estimated from all the negative controls in the experiment *j*:

$\hat{b_j}=\mathrm{median}\ \Big(\ln \left({C}_{kj}/{S}_{kj}\right)$) for *k* = 1, …, *K* in *K* negative control sgRNAs.

With *d*_*ij*_ = $\ln \left({C}_{ij}/{S}_{ij}\right)-\hat{b_j}$, we have

2 $$ {d}_{ij}=\mathit{\ln}\left(1+{p}_{ij}\left({e}^{f_{ij}}-1\right)\right) $$

here 0 < *p*_*ij*_ < 1, when ${p}_{ij}\left({e}^{f_{ij}}-1\right)$ is small, $\ln \left(1+{p}_{ij}\left({e}^{f_{ij}}-1\right)\right)$ is approximately equal to ${p}_{ij}\left({e}^{f_{ij}}-1\right).$ Thus we have:

3 $$ {d}_{ij}={p}_{ij}\left({e}^{f_{ij}}-1\right) $$

Here the percentage of cells with the variant *i p*_*ij*_ can be described by

$$ {p}_{ij}={q}_i{l}_j $$

where *q*_*i*_ is the relative activity of sgRNA *i* for generating the variant *i*, which can be measured separately from our editing efficiency experiment *j*. *l*_*j*_ is the efficiency term determined by factors other than sgRNA target site sequence in the experiments, such as the base editor expression level and base editor activity. For the same screen, *l*_*j*_ is equal for all the sgRNAs used in the screen. For our analysis, *l*_*j*_ is typically not estimated and denotes an experiment-specific constant.

Then we define the functional score *β*_*ij*_ for variant *i* as ${\beta}_{ij}={l}_j\left({e}^{f_{ij}}-1\right)$. We have the following formula for calculating the functional score *β*_*ij*_:

4 $$ {d}_{ij}={q}_i{\beta}_{ij} $$

This linear relationship implies that *d*_*ij*_ is also proportional to ${e}^{f_{ij}}$, which in turn depends on the approximation in (3). Our data is largely consistent with the linear relationship and not sensitive to departure from the approximation in (3) (see “Results”).

### Calculating functional scores for variants with editing efficiency correction

To perform editing efficiency correction with the model, the following steps were taken: First, sgRNAs having editing outcome with coverage less than 30 reads or frequency below 0.005 in both replicates of the genome-integrated target site assay were removed. Second, the average efficiency of the two replicates for each editing outcome was calculated as the measured efficiency. Third, the outcome and corresponding efficiency of all sgRNAs were predicted with BE-Hive [25]. Fourth, the BE-Hive predicted and measured efficiency of editing outcomes were then quantile normalized to each other, and the corrected measured efficiency value was assigned to each editing outcome. For the outcome with no measured efficiency, the BE-Hive predicted efficiency value was assigned. Fifth, the efficiency values for all the editing outcome were linearly scaled to the range of 0 to 1. Finally, the β score was calculated according to formula (4).

### Identification of loss-of-function variants using Gaussian mixture model

A four-component Gaussian mixture model was used to fit the distribution of functional scores for classification of loss-of-function variants. The mixture.GaussianMixture function in the Python package “sklearn” was used to train the model, and the parameter “n_components” was set to 4. The fitted model consisted of four Gaussian nuclei, one of which had a mean value near 0, two of which had negative mean values, and one had positive mean value. The summed probability of a variant *i* with the score *β*_*i*_ been classified to any of the two distributions that having negative mean value was calculated as *P*_*i*_.

The Wald test was used to calculate the significance of *β* scores, by comparing the value of $\frac{\mathrm{mean}\ \mathrm{of}\ \beta\ \mathrm{score}}{\mathrm{standard}\ \mathrm{error}\ \mathrm{of}\ \beta\ \mathrm{score}}$ to a standard Normal distribution. The *p* values were corrected with the Benjamini & Hochberg method [52].

## Supplementary Information

**Additional file 1: Figure S1.** Validation of BRCA1/2 essentiality in eHAP cells. **Figure S2.** Quality control plots for base editing screens. **Figure S3.** Genome-integrated target site library assay for determining editing activities of sgRNAs. **Figure S4.** Comparison of predicted base editing activities with measured activities. **Figure S5.** Gaussian mixture modeling of functional scores for the screens. **Figure S6.** Quality control plots for sgRNA editing activity mapping experiments with two “NGN” recognizing base editors. **Figure S7.** Cell viability analysis of eHAP cells after inducing indicated mutations in 5’ UTR regions with indicated sgRNAs. **Figure S8.** Gaussian mixture modeling of functional scores with different numbers of components.

**Additional file 2: Table S1.** Detailed data of CCK-8 growth assay and qPCR for Figure S1a-d, Fig. 4h, Fig. 6h,i and Figure S6a-b.

**Additional file 3: Table S2.** Sequences of sgRNAs and MAGeCK scores for BRCA1/2-NGG and BRCA1/2-NG screens.

**Additional file 4: Table S3.** Oligos used in the integrated-genome target site library assay.

**Additional file 5: Table S4.** Distribution of the number of outcomes for sgRNAs after editing with AncBE4max or ABEmax, before and after merging outcomes at amino acid level. Related to Fig. 2c.

**Additional file 6: Table S5.** Outcome mapping of sgRNAs using genome-integrated target site library assay.

**Additional file 7: Table S6.** Classifications of variants for BRCA1/2 screens.

**Additional file 8: Table S7.** Overlap of loss-of-function variants with other studies.

**Additional file 9: Table S8.** Overlap of LOF variants in the NGG screen and the NG screen.

**Additional file 10: Table S9.** Sequences of PCR oligos and sgRNAs used in this study.

**Additional file 11: Table S10.** AIC and BIC values for Gaussian mixture models with different numbers of components.

**Additional file 12: Table S11.** AUC values for different classification models with different parameters.

**Additional file 13: Supplementary Note 1.**

**Additional file 14.** Review History.

## Data Availability

The datasets and materials generated during the current study are available on the NCBI BioProject Archive under accession no. PRJNA656176 [53]. The code repository for data analysis about this study is available at https://github.com/guangyu1990/Edit_Efficiency_Correction, with license GPL-3.0. Source code used in the manuscript is available via Zenodo with DOI 10.5281/zenodo.4553101 [54].

## References

[1] Bailey MH, et al. Comprehensive characterization of Cancer driver genes and mutations. Cell. 2018;173(2):371–85. e18 10.1016/j.cell.2018.02.060 PMC6029450 29625053

[2] Richards S (2015). Standards and guidelines for the interpretation of sequence variants: a joint consensus recommendation of the American College of Medical Genetics and Genomics and the Association for Molecular Pathology. Genet Med.

[3] Roy R, Chun J, Powell SN (2011). BRCA1 and BRCA2: different roles in a common pathway of genome protection. Nat Rev Cancer.

[4] Landrum MJ (2014). ClinVar: public archive of relationships among sequence variation and human phenotype. Nucleic Acids Res.

[5] Bouwman P (2013). A high-throughput functional complementation assay for classification of BRCA1 missense variants. Cancer Discov.

[6] Ransburgh DJ (2010). Identification of breast tumor mutations in BRCA1 that abolish its function in homologous DNA recombination. Cancer Res.

[7] Starita LM (2018). A multiplex homology-directed DNA repair assay reveals the impact of more than 1,000 BRCA1 missense substitution variants on protein function. Am J Hum Genet.

[8] Findlay GM (2014). Saturation editing of genomic regions by multiplex homology-directed repair. Nature.

[9] Wang H (2013). One-step generation of mice carrying mutations in multiple genes by CRISPR/Cas-mediated genome engineering. Cell.

[10] Ranjha L, Howard SM, Cejka P (2018). Main steps in DNA double-strand break repair: an introduction to homologous recombination and related processes. Chromosoma.

[11] Findlay GM (2018). Accurate classification of BRCA1 variants with saturation genome editing. Nature.

[12] Haapaniemi E (2018). CRISPR-Cas9 genome editing induces a p53-mediated DNA damage response. Nat Med.

[13] Allen F, Crepaldi L, Alsinet C, Strong AJ, Kleshchevnikov V, De Angeli P, et al. Predicting the mutations generated by repair of Cas9-induced double-strand breaks. Nat Biotechnol. 2018; 10.1038/nbt.4317 10.1038/nbt.4317 PMC6949135 30480667

[14] Komor AC (2016). Programmable editing of a target base in genomic DNA without double-stranded DNA cleavage. Nature.

[15] Gaudelli NM (2017). Programmable base editing of A*T to G*C in genomic DNA without DNA cleavage. Nature.

[16] Komor AC (2017). Improved base excision repair inhibition and bacteriophage Mu Gam protein yields C:G-to-T:A base editors with higher efficiency and product purity. Sci Adv.

[17] Koblan LW (2018). Improving cytidine and adenine base editors by expression optimization and ancestral reconstruction. Nat Biotechnol.

[18] Nishimasu H (2018). Engineered CRISPR-Cas9 nuclease with expanded targeting space. Science.

[19] Huang TP (2019). Circularly permuted and PAM-modified Cas9 variants broaden the targeting scope of base editors. Nat Biotechnol.

[20] Thuronyi BW (2019). Continuous evolution of base editors with expanded target compatibility and improved activity. Nat Biotechnol.

[21] Kweon J (2020). A CRISPR-based base-editing screen for the functional assessment of BRCA1 variants. Oncogene.

[22] Despres PC (2020). Perturbing proteomes at single residue resolution using base editing. Nat Commun.

[23] Hanna RE, Doench JG (2020). Design and analysis of CRISPR-Cas experiments. Nat Biotechnol.

[24] Sakata RC, et al. Base editors for simultaneous introduction of C-to-T and A-to-G mutations. Nat. Biotechnol. 2020;38(7):865–69. 10.1038/s41587-020-0509-0 32483365

[25] Arbab M, et al. Determinants of base editing outcomes from target library analysis and machine learning. Cell. 2020;182(2):463–80. 10.1016/j.cell.2020.05.037 PMC7384975 32533916

[26] Blomen VA (2015). Gene essentiality and synthetic lethality in haploid human cells. Science.

[27] Grillo G (2010). UTRdb and UTRsite (RELEASE 2010): a collection of sequences and regulatory motifs of the untranslated regions of eukaryotic mRNAs. Nucleic Acids Res.

[28] Smith CJ (2020). Enabling large-scale genome editing at repetitive elements by reducing DNA nicking. Nucleic Acids Res.

[29] Kim HK (2017). In vivo high-throughput profiling of CRISPR-Cpf1 activity. Nat Methods.

[30] Wang D (2019). Optimized CRISPR guide RNA design for two high-fidelity Cas9 variants by deep learning. Nat Commun.

[31] Zhang X (1998). Structure of an XRCC1 BRCT domain: a new protein-protein interaction module. EMBO J.

[32] Yang H (2002). BRCA2 function in DNA binding and recombination from a BRCA2-DSS1-ssDNA structure. Science.

[33] Guidugli L (2013). A classification model for BRCA2 DNA binding domain missense variants based on homology-directed repair activity. Cancer Res.

[34] Gruber AR, Bernhart SH, Lorenz R (2015). The ViennaRNA web services. Methods Mol Biol.

[35] Jia L (2020). Decoding mRNA translatability and stability from the 5′ UTR. Nat Struct Mol Biol.

[36] Schuster SL, Hsieh AC (2019). The untranslated regions of mRNAs in cancer. Trends Cancer.

[37] Song M, et al. Sequence-specific prediction of the efficiencies of adenine and cytosine base editors. Nat. Biotechnol. 2020;38(9):1037–43. 10.1038/s41587-020-0573-5 32632303

[38] Anzalone AV, Koblan LW, Liu DR (2020). Genome editing with CRISPR–Cas nucleases, base editors, transposases and prime editors. Nat Biotechnol.

[39] Daley TP (2018). CRISPhieRmix: a hierarchical mixture model for CRISPR pooled screens. Genome Biol.

[40] Grunewald J (2019). Transcriptome-wide off-target RNA editing induced by CRISPR-guided DNA base editors. Nature.

[41] Zhou C (2019). Off-target RNA mutation induced by DNA base editing and its elimination by mutagenesis. Nature.

[42] Zuo E (2019). Cytosine base editor generates substantial off-target single-nucleotide variants in mouse embryos. Science.

[43] Anzalone AV (2019). Search-and-replace genome editing without double-strand breaks or donor DNA. Nature.

[44] Sanjana NE, Shalem O, Zhang F (2014). Improved vectors and genome-wide libraries for CRISPR screening. Nat Methods.

[45] Bae S, Park J, Kim JS (2014). Cas-OFFinder: a fast and versatile algorithm that searches for potential off-target sites of Cas9 RNA-guided endonucleases. Bioinformatics.

[46] Bolger AM, Lohse M, Usadel B (2014). Trimmomatic: a flexible trimmer for Illumina sequence data. Bioinformatics.

[47] Li H, Durbin R (2009). Fast and accurate short read alignment with Burrows-Wheeler transform. Bioinformatics.

[48] Xu L, Liu Y, Han R (2019). BEAT: a Python program to quantify base editing from sanger sequencing. CRISPR J.

[49] Li W (2014). MAGeCK enables robust identification of essential genes from genome-scale CRISPR/Cas9 knockout screens. Genome Biol.

[50] Fu L (2012). CD-HIT: accelerated for clustering the next-generation sequencing data. Bioinformatics.

[51] Wang K, Li M, Hakonarson H (2010). ANNOVAR: functional annotation of genetic variants from high-throughput sequencing data. Nucleic Acids Res.

[52] Benjamini Y, Hochberg Y (1995). Controlling the false discovery rate: a practical and powerful approach to multiple testing. J R Stat Soc Series B (Methodological).

[53] Huang CC, Li GY, Wu JY, Liang JB, Wang XY. Identification of pathogenic variants in cancer genes using base editing screens with editing efficiency correction. Datasets. National Center for Biotechnology Information. https://www.ncbi.nlm.nih.gov/bioproject/656176 (2020). 10.1186/s13059-021-02305-2 PMC7945310 33691754

[54] Huang CC, Li GY, Wu JY, Liang JB, Wang XY. Identification of pathogenic variants in cancer genes using base editing screens with editing efficiency correction*.* Zenodo. 10.5281/zenodo.4553101 (2020). 10.1186/s13059-021-02305-2 PMC7945310 33691754

