# Supplementary material for: Identification of pathogenic variants in cancer genes using base editing screens with editing efficiency correction
Source: Genome Biol. 2021 Mar 10;22:80. doi: 10.1186/s13059-021-02305-2 (PMC7945310; doi:10.1186/s13059-021-02305-2)
Supplement: Supplementary file 1 — Additional file 1: Figure S1. Validation of BRCA1/2 essentiality in eHAP cells. Figure S2. Quality control plots for base editing screens. Figure S3. Genome-integrated target site library assay for determining editing activities of sgRNAs. Figure S4. Comparison of predicted base editing activities with measured activities. Figure S5. Gaussian mixture modeling of functional scores for the screens. Figure S6. Quality control plots for sgRNA editing activity mapping experiments with two “NGN” recognizing base editors. Figure S7. Cell viability analysis of eHAP cells after inducing indicated mutations in 5’ UTR regions with indicated sgRNAs. Figure S8. Gaussian mixture modeling of functional scores with different numbers of components. [file 13059_2021_2305_MOESM1_ESM.docx]

Supplementary figures


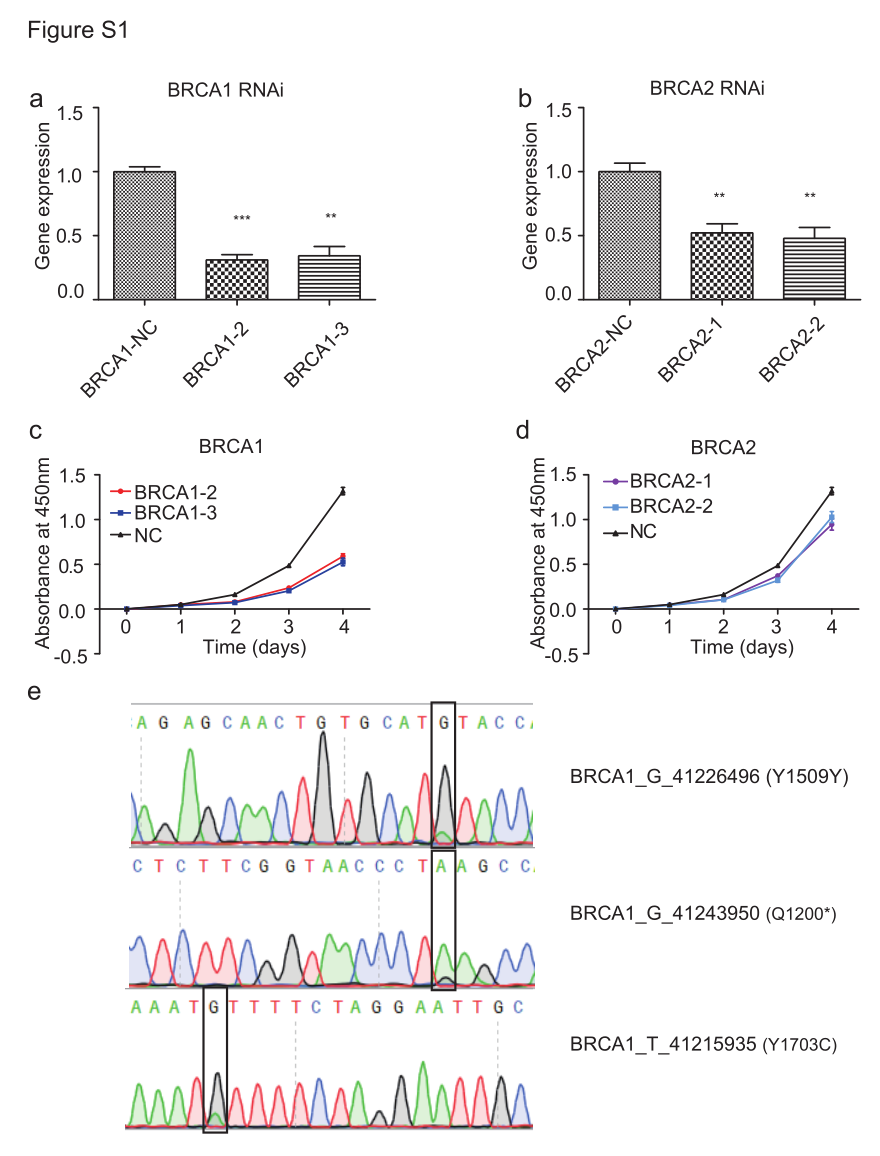


**Figure S1. Validation of BRCA1/2 essentiality in eHAP cells.**

**a,b** Gene expression analysis after knocking down *BRCA1* (**a**) or *BRCA2* (**b**) using RNAi. Expression levels of *BRCA1* or *BRCA2* in treated groups were normalized to non-targeting control groups. Error bars represent SEMs from 3 independent quantitative PCR experiments and two-tailed Student’s t tests were used to determine *P* values. **c**,**d** Cell viability analysis of eHAP cells for four days using CCK-8 assay after transfection of siRNAs targeting *BRCA1* (**c**) or *BRCA2* (**d**). Three experiments were performed and error bars represent SEMs. **e** Sanger sequencing traces showing the base editing results for three sgRNAs as indicated. Genomic DNAs were collected from eHAP cells and PCR amplified for sequencing after 48 hours of base editor transfection. For all graphs, unpaired and two-tailed *t*-tests were used to calculate *P*-values. ***P* < 0.01; ****P* < 0.001.


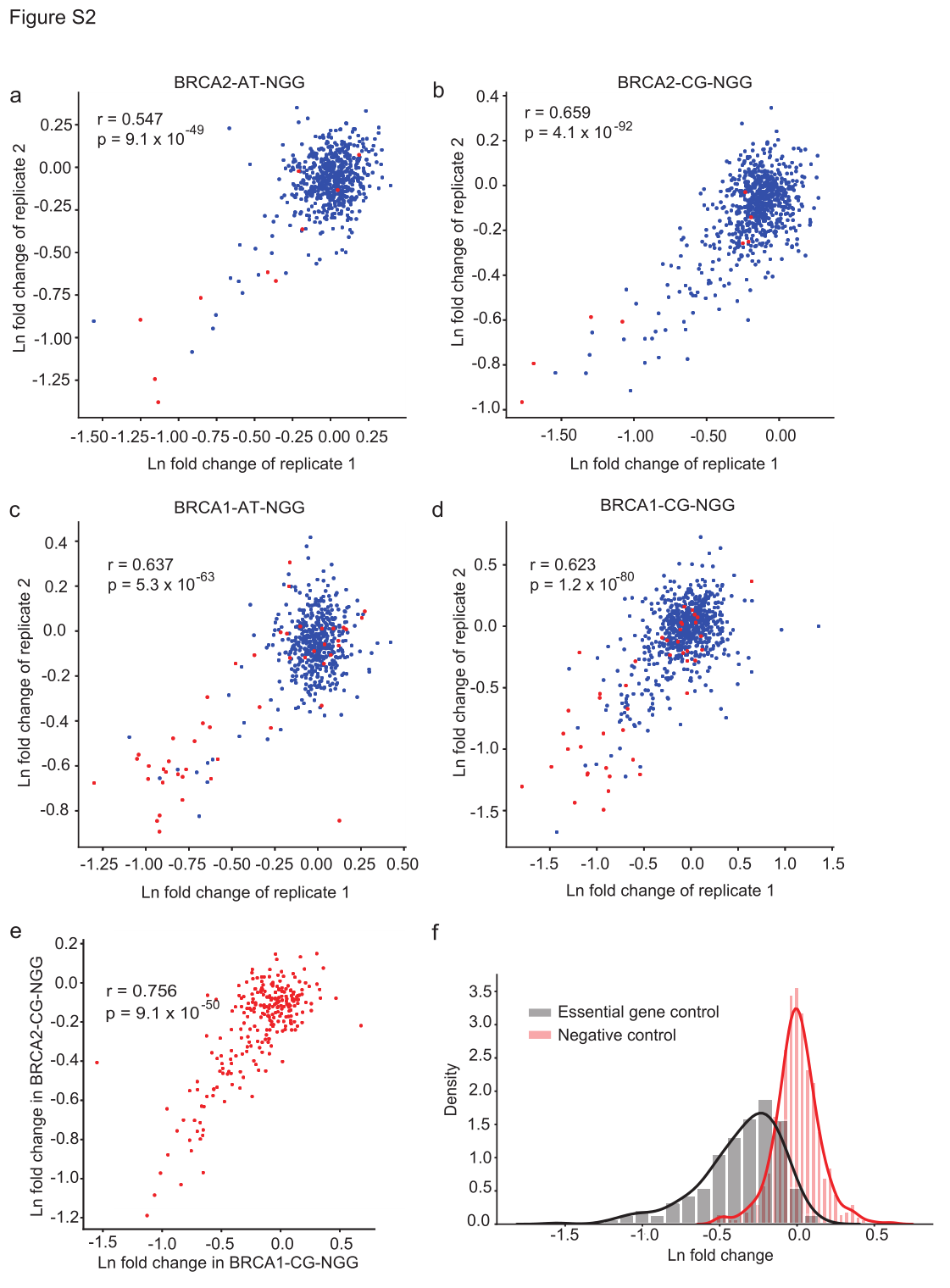


**Figure S2. Quality control plots for base editing screens.**

**a-d** Scatterplots showing replicates comparison for ln fold change of normalized sgRNA read counts in four base editing screens: BRCA2-AT-NGG (**a**), BRCA2-CG-NGG (**b**), BRCA1-AT-NGG (**c**), and BRCA1-CG-NGG (**d**). Red dots represent sgRNAs that targeting more than three sites in the genome. Pearson’s correlation coefficients (r) are shown. **e** Ln fold change of essential gene control sgRNAs in BRCA1-CG-NGG and BRCA2-CG-NGG screens. Pearson’s correlation coefficient (r) is shown. **f** Histogram and kernel density estimate of ln fold change for essential gene control and negative control sgRNAs. The fitted kernel density lines were estimated by ‘displot’ in Python package ‘seaborn’ with the default parameter.


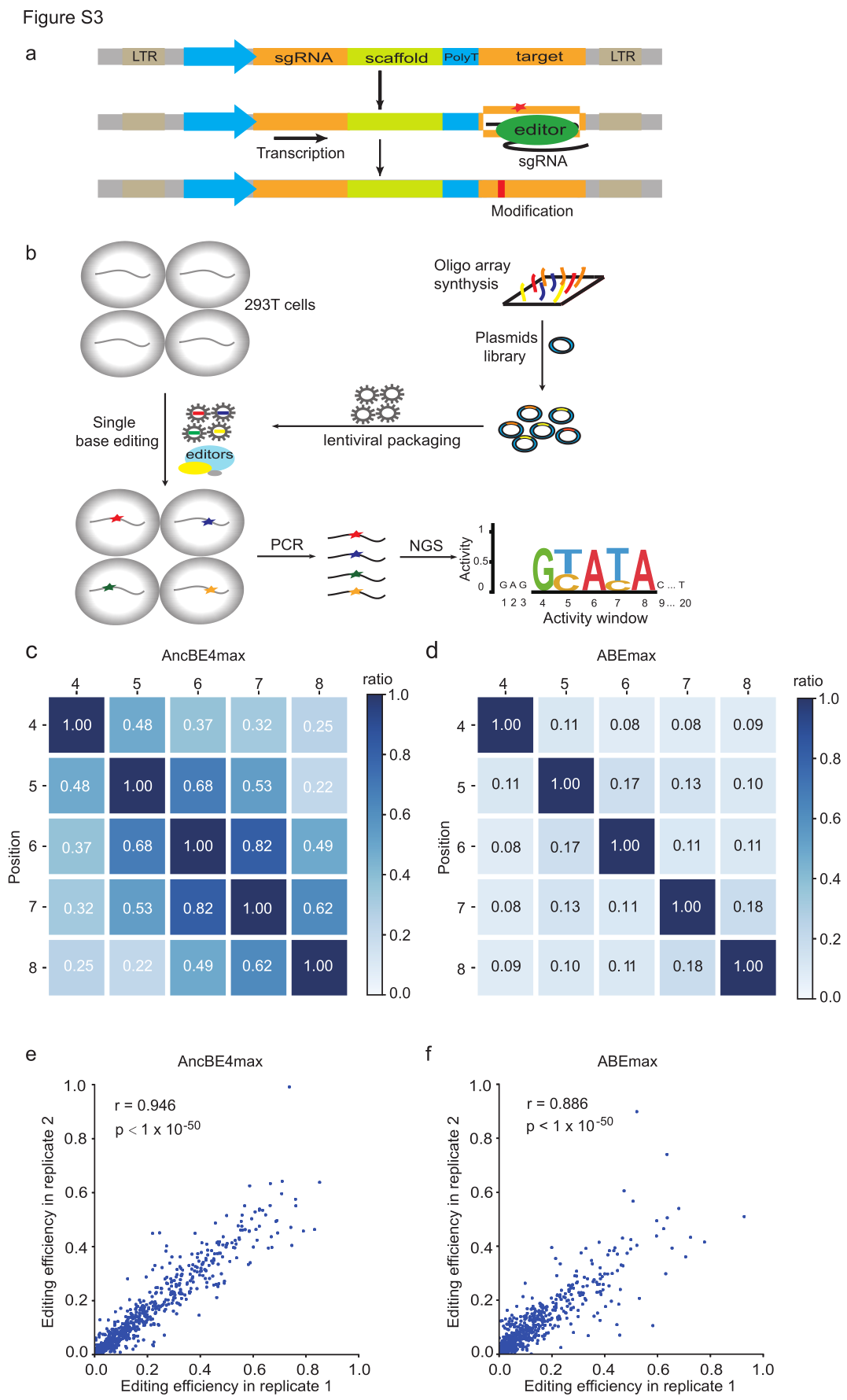


**Figure S3. Genome-integrated target site library assay for determining editing activities of sgRNAs.**

**a** Schematic diagram of the vector containing the sgRNA-target pair. Blue arrow represents the human U6 promoter and other components are ordered as labeled. The target site consists of three nucleotides upstream of the sgRNA complementary sequence and PAM sequence. **b** Schematic overview of the genome-integrated target site library assay for measuring sgRNA activities. Oligo array containing the designed sgRNA-target pairs were cloned into lentiguide-puro vectors, packaged into lentivirus and infected into cells. After selecting by puromycin, the cells were transfected by base editors for 48 h. The cells were selected for EGFP expression by FACS. Editing efficiencies of sgRNAs were calculated by sequencing the sgRNA-target cassettes. **c,d** Average co-editing frequencies of nucleotides based on their protospacer positions in the editing window of AncBE4max (**c**) or ABEmax (**d**). The co-editing efficiencies were normalized to a maximum of 100%. **e,f** Correlations of editing activity at tested target sites between replicates for AncBE4max (**e**) or ABEmax (**f**). Pearson’s correlation coefficients (r) are shown.


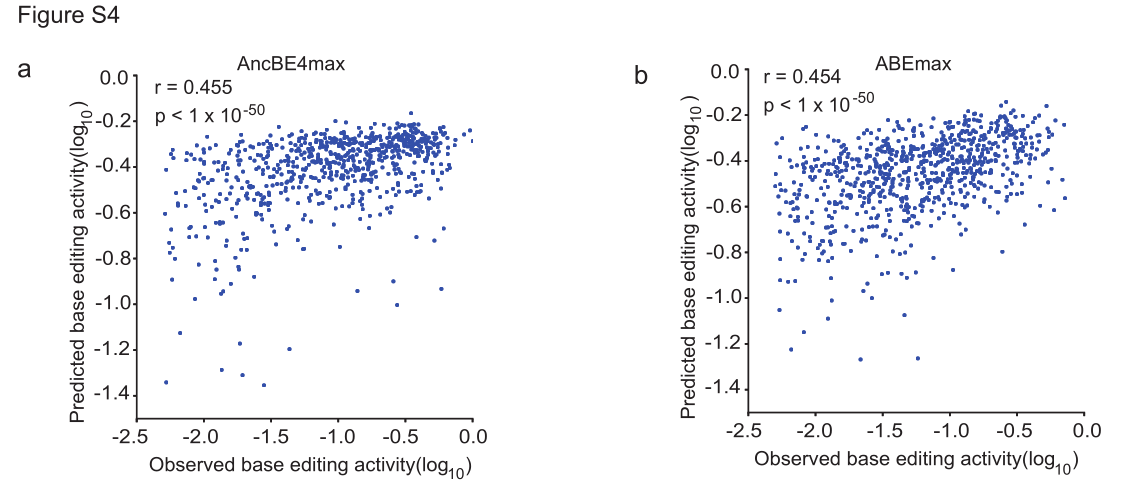


**Figure S4** **Comparison of predicted base editing activities with measured activities.**

**a,b** Correlations of observed and BE-Hive predicted editing activity for AncBE4max (**a**) or ABEmax (**b**). r represents Pearson’s correlation coefficient.


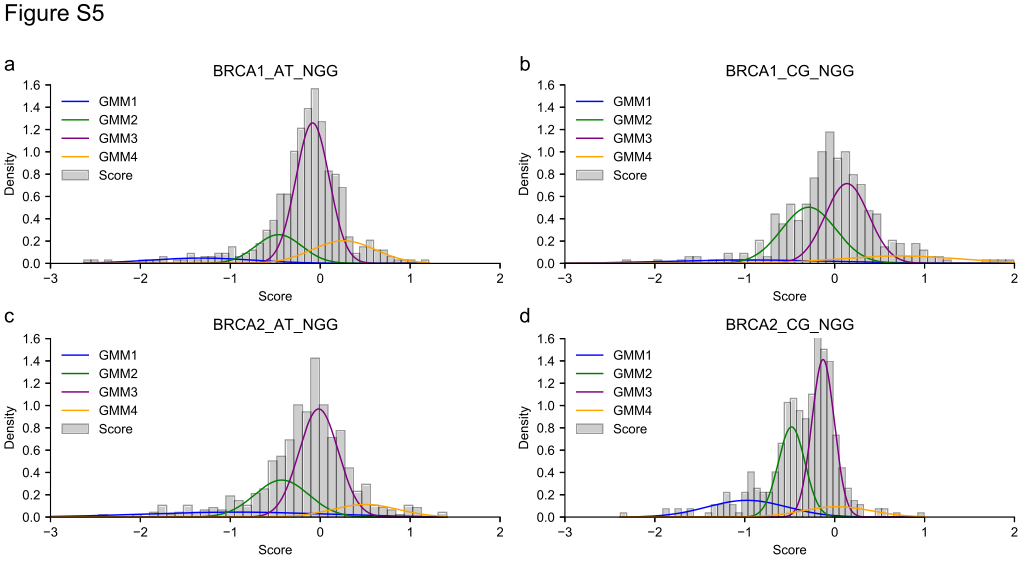


**Figure S5 Gaussian mixture modeling of functional scores for the screens. a-d** Gaussian mixture model of 4 normal distributions for BRCA1-AT-NGG (**a**), BRCA1-CG-NGG (**b**), BRCA2-AT-NGG (**c**), and BRCA2-CG-NGG (**d**).


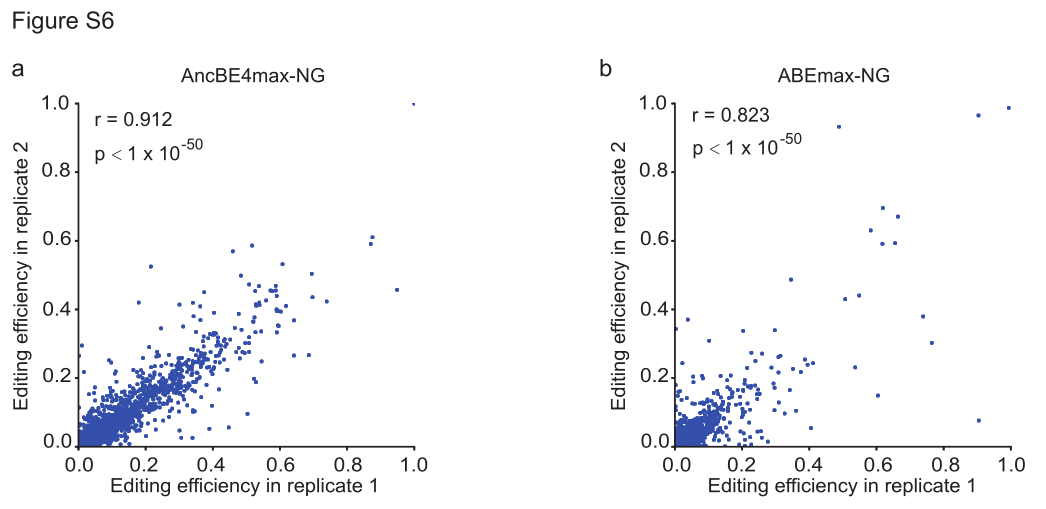


**Figure S6 Quality control plots for sgRNA editing activity mapping experiments with two “NGN” recognizing base editors.**

**a,b** Correlations of editing activity at tested target sites between replicates for AncBE4max-NG (**a**) or ABEmax-NG (**b**). Pearson’s correlation coefficients (r) are shown.


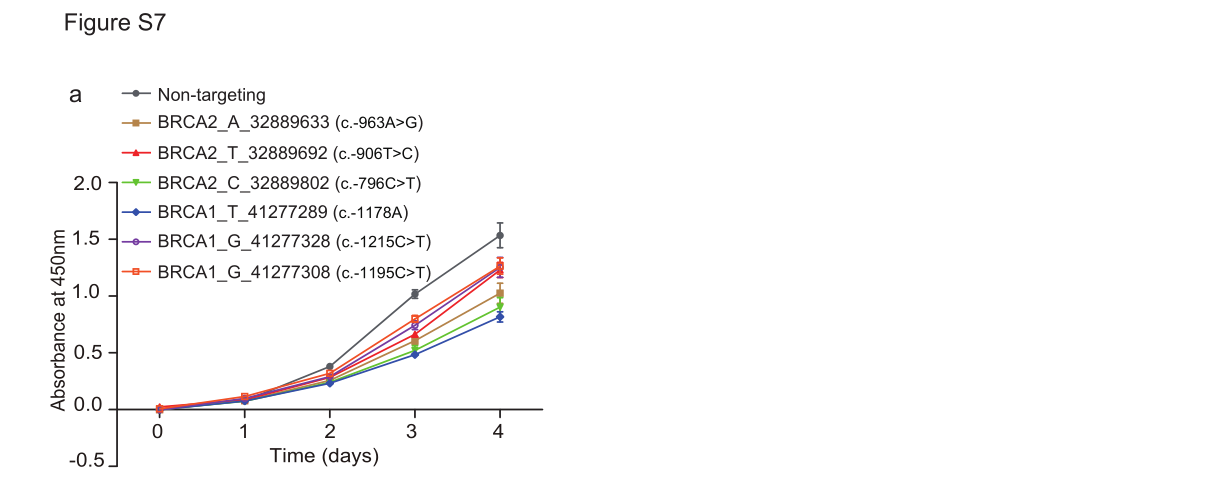


**Figure S7**. **Cell viability analysis of eHAP cells after inducing indicated mutations in 5’ UTR regions with indicated sgRNAs.** Cell viability were measured for four days using CCK-8 assay. Detailed data are listed in Table S1.


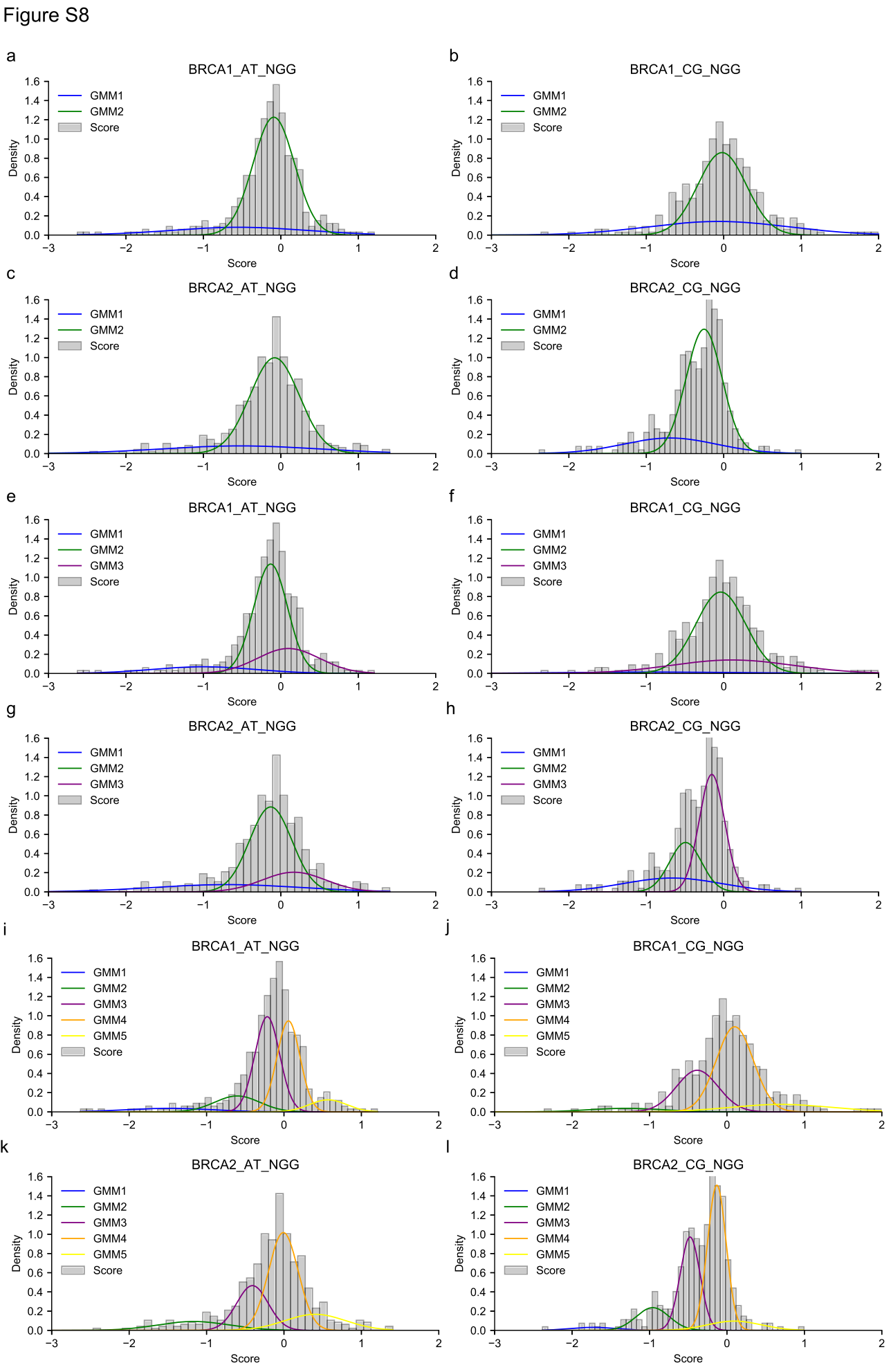


**Figure S8 Gaussian mixture modeling of functional scores with different numbers of components. a-d** Gaussian mixture model of 2 normal distributions for BRCA1-AT-NGG (**a**), BRCA1-CG-NGG (**b**), BRCA2-AT-NGG (**c**), and BRCA2-CG-NGG (**d**). **e**-**h** Gaussian mixture model of 3 normal distributions for BRCA1-AT-NGG (**e**), BRCA1-CG-NGG (**f**), BRCA2-AT-NGG (**g**), and BRCA2-CG-NGG (**h**). **i**-**l** Gaussian mixture model of 5 normal distributions for BRCA1-AT-NGG (**i**), BRCA1-CG-NGG (**j**), BRCA2-AT-NGG (**k**), and BRCA2-CG-NGG (**l**).
