# Supplementary material for: Identification of pathogenic variants in cancer genes using base editing screens with editing efficiency correction
Source: Genome Biol. 2021 Mar 10;22:80. doi: 10.1186/s13059-021-02305-2 (PMC7945310; doi:10.1186/s13059-021-02305-2)
Supplement: Supplementary file 13 — Additional file 13: Supplementary Note 1. [file 13059_2021_2305_MOESM13_ESM.docx]

Supplementary Note 1

To select the Gaussian mixture model that best fit the data, we applied the models with component 2,3,4,5,6 on each screen. From the AIC or BIC scores, the models with the smallest AIC or BIC scores differed between different screens (Supplementary Table S10 and Supplementary Figure S8). We tried different classification strategies based on combinations of different mixture models and different parameters. Using the known pathogenic and benign variants to evaluate the performance of each model, we found the four-component model achieved the highest AUC value for identification of LOF variants (Supplementary Table S11). Based on AUC values, we also found that the best criteria to classify the loss-of-function (LOF) variants was to use the probability (P_i_) that a variant i belonging to any of the two distributions with negative means greater than 0.8 for NGG screens.

Similarly, for the NG screens, although the AIC/BIC values were optimal for the 2-component model for all the four libraries, we found that applying four-component Gaussian mixture model to all the screens achieved the highest AUC score (Supplementary Table 11). And the optimal parameter was P_i_>0.9 for NG screens.

We estimated the FDR using p-values for $\beta$ scores calculated from the Wald test, by comparing the value of $\frac{mean of \beta\mathrm{score}}{standard error of \beta\mathrm{score}}$ to a standard Normal distribution (Supplementary Table S6). The LOF variants were all under an FDR of 0.2 for all NGG screens and an FDR of 0.1 for all NG screens.
