# Supplementary material for: Identification of pathogenic variants in cancer genes using base editing screens with editing efficiency correction
Source: Genome Biol. 2021 Mar 10;22:80. doi: 10.1186/s13059-021-02305-2 (PMC7945310; doi:10.1186/s13059-021-02305-2)
Supplement: Supplementary file 14 — Additional file 14. Review History. [file 13059_2021_2305_MOESM14_ESM.docx]

Review History

**First round of review**

**Reviewer 1**

**Are you able to assess all statistics in the manuscript, including the appropriateness of statistical tests used?** Yes, and I have assessed the statistics in my report.

**Comments to author:**

CRISPR-based base editors, including CBE, ABE and recently developed CGBE system, can introduce C-to-T, A-to-G and C-to-G substitutions in eukaryotic genome target sites, respectively, providing a new strategy to perform the high-throughput screening in genome-wide or specific genomic regions. Previous studies have reported that the original version of cytosine base editor, BE3, could be used to screen for the functional assessment of BRCA1 variants. In this manuscript, Huang et al. developed an optimized framework for base editor-based screen with AncBE4max (CBE) and ABEmax (ABE) in BRCA1 and BRCA2 genes.

Major Comments:

1. The manuscript focused on improving the base editing screening method by developing a score model, but the authors did not directly compare the new screening method to the old sgRNA fold-change-based screening methods. I can see that the new method needs more experimental data than the old methods, such as the integrated-genome target library assay. The authors should clearly confirm that the new method has higher screening efficiency or accuracy than the old methods.

2. The authors identified the loss-of-function BRCA1 variants and 530 BRCA2 variants, and validated four sgRNAs using cell viability assays. These four sgRNAs resulted in the delayed growth of eHAP cells compared with non-targeting controls (Fig 6H). However, the control sgRNAs that targeted nonsense mutations should also be tested to evaluate the accuracy of this method. The score of tested sgRNAs should be correlated with their degrees of delayed growth of eHAP.

3. The complexity of editing outcome is one of the major issues the authors should focus on. In Line 202-203, the authors defined the editing outcome for each sgRNA as the editing result with the highest frequency. The average co-editing frequencies of nucleotides based on their protospacer positions in the editing window of AncBE4max or ABEmax were analyzed in Fig 2 C/D, but the authors did not directly analyze the purity of editing products for each sgRNA. The editing outcome complexity for each sgRNA may still cause perturbations in the model. Moreover, in Line 198, why did the authors analyze "two consecutively Cs or As"? The editing products of AncBE4max and ABEmax base editor are influenced by the chromatin environment, catalytic activity of deamination, editing preference and so on. Analyzing the co-editing frequencies could not reflect that "AncBE4max or ABEmax only produce one type of products".

4. Spearman's correlation coefficient was used to calculate the correlation between the results, which is a method of evaluating the degree of correlation based on ranking data. But this method is not suitable for this study. Other methods should be used, such as Pearson's correlation coefficient. In addition, no matter which method is used, the definition of "good correlations" in this manuscript seems to be too loose. "good correlations" appears many times in the manuscript, but the minimum value of "good correlations" is only 0.466. More statistical methods should be provided to prove the reliability of the results.

5. In Lines 226-229: the authors stated that "As displayed by simulated data, within conditions of our experiments, we found the natural logarithm (ln) fold change of a sgRNA was almost linearly correlated with the editing activity of the sgRNA". The editing efficiency itself is not high and it likely causes many errors. Besides, it is also found in Fig 2B that it did not conform to a normal distribution or an approximate normal distribution. There was no sufficient evidence to prove this conclusion. It was not correct to use the linear model directly.

6. The results of Fig4 E/F (NGG) and Fig5 E/F (NG) were quite different, especially in the "Stop gain" group. The authors have to clarify it. Is it because of the different library content of NG and NGG?

Minor Comments:

1. Line 111-113: "In a pooled screen, the change of sgRNA abundance between two conditions is often used to evaluate the effects of sgRNAs". Related reference should be cited here.

2. Line 141-158: what are the reasons for choosing these three sgRNAs? Why not repeat the same test in BRCA2 gene region? The sequences and other detailed information of selected three sgRNA are missing.

3. Line 159: "1,908 sgRNAs", only 728 sgRNAs were found in Table S1.

4. Line 162-163: Related sequences or other detailed information about non-targeting sgRNAs are missing.

5. Line 176-177: Related sequences or other detailed information about 47 sgRNAs targeting SINE/Alu are missing.

6. Line 177: Please explain how to determine SINE/Alu repeats and cite related references.

7. Line 191-193: The explanation of orange dash is missing in figure legend of Fig. 2B. Is it average or median?

8. Line 270-274: "These results suggested the assay was able to provide functional assessment for variants in both coding and noncoding regions of genes". Please clarify it.

9. Line 468: "a 82 bp protospacer" should be "an 82 bp sgRNA Scaffold".

10. Fig 1B: In Fig 1B, different categories are difficult to distinguish; same problem in Fig S1. More colors could be used.

11. Table S1: What is the meaning of "C-G_NGG" in lib_type? it looks like GBE (C:G to G:C conversion) library.

12. The significance test method used in this study should be given.

13. Many details about the positive and negative controls are missing in this manuscript and some figures could be moved to Supplementary Materials.

**Reviewer 2**

**Are you able to assess all statistics in the manuscript, including the appropriateness of statistical tests used?** Yes, and I have assessed the statistics in my report.

**Comments to author:**

In "Identification of pathogenic variants in cancer genes using base editing screens with efficiency correction", Huang & Li et al leverage base editors to investigate the effect of thousands of mutations in the BRCA1 and BRCA2 genes. They perform 8 different screens (2 BEs x 2 genes x 2 Cas9) to enhance the number of variants characterized, while also performing large-scale editing efficiency testing of their gRNAs in parallel. They then use this data to develop a model that adjusts the essentiality scores to take into account the intrinsic efficiency of each gRNA.

Overall, I found the manuscript to be well written and interesting. I particularly enjoyed the idea of performing fitness assays in parallel with editing efficiency assays, which has the potential to address an important bias in large-scale base editing applications. I think the content of the manuscript is of interest to many in the genome editing community as well as those interested in genotype-to-phenotype maps. That being said, I think the manuscript needs much improvement before it is ready for publication. In particular, I found a lot of data to be missing and that multiple aspects of the method could be better explained. Here are my comments:

1) There have been multiple studies that used recent advances in genome editing to characterize the effect of BRCA1 variants, including one that used the BE3 base editor in HAP1 cells. While the authors cite these studies (Findlay et al 2018 and Kweon et al 2020), they should also compare their results and discuss whether or not there is a significant overlap in the loss of function (LOF) mutants they observe. Furthemore, the authors should cite Findlay et al 2018 as well Blomen et al 2015 (Science) when discussing the cell growth phenotypes of LOF mutants, as these genes had already been shown to be essential in Haploid human cells.

2) In multiple parts of the manuscript, there is not enough information on the statistical tests or observations performed. For example, no p-values are provided for any of the correlations presented in the main or supplementary figures.

3) The authors should discuss why there seems to be an important difference in replicate correlation between the AT and CG screens (as shown in figure S2)

4) Based on the information provided in the text and in Figure 2, I worry that the sequencing coverage used in the genomic integration BE assay is too low to accurately measure which editing outcomes are the most frequent. As stated on line 192, the median target site coverage for each gRNA was 4000 reads. If we consider a median editing rate of 4.6% (as stated on line 193), this would mean that the "editing activity" was often calculated based on very few reads (e.g. : a gRNA with an editing rate of 2% and a coverage of 2000 reads = 40 reads). It might be useful to add minimal depth thresholds for editing "editing activity" determination so that only gRNAs with sufficient coverage are kept. This bias would also affect the co-editing efficiency analysis. I saw that there is a 30 reads or 0.005 frequency cut-off in the "editing efficiency correction" section (703-704), but it is unclear how this is taken into account in the rest of the manuscript.

5) Related to this issue, there is not enough information available on the integrated target genome site library assay for the NG editor libraries. Was the median coverage identical to the NGG editor libraries? How well did the different replicates correlate?

6) I have some interrogations on the data presented in figure 2 E and F. First, both axes of both panels should be scaled identically, and in a more intuitive way (e.g.: higher editing rates = higher value on the axis). Second, If I understood correctly lines 587-588 in the methods, gRNAs for which no editing could be observed were excluded from this figure and the correlation coefficient calculations? If so, could the authors justify this omission? It seems like important information to know that a subset of gRNAs did not work at all in the eHAP cell lines.

7) The same comment applies to panels B and C of figure 3. The y axis should have the same scaling.

8) The same comment applies to Figure 4 panels A, B, C and D. The y-axis should be scaled based on density, not absolute numbers, and the histogram bin size should be the across all categories and panels.

9) Finally, this comment also applies to figure 5 C, D and E, F, G, H. The axis for the scatter plots shown in figure 5 C and D should be scaled the same way. For panels E, F, G, H, the y-axis should be scaled based on density, not absolute numbers, and the histogram bin size should be the across all categories and panels.

10) The authors should provide the beta score data for the NG editor screens, not just for the NGG editor experiments.

11) The methods section on the Gaussian mixture modeling is severely lacking in detail. While this can be partially addressed by making the computer code that underlie it open access, there should more information on how the data was processed. The authors should provide statistical support for their 4 nuclei model and provide the AIC values associated with the different hypothesis they tested.

12) As this manuscript contains a lot of computational work and presents a new method for large-scale BE scree data analysis, all the codes used should be made available. This includes both the code used to perform the data analysis and to generate the figures as well as the sequencing analysis pipelines.

13) I think the manuscript would be much improved by additional validation experiments and follow up on screen hits. Furthermore, I think the validation experiments would be stronger if the mutant cell lines were not generated by BE but by other another editing approach. Having some mutant specific assays (such as expression measurements for the UTR mutations) would also be a plus.

14) Finally, I think that while the editing efficiency correction idea is splendid, the authors do not show its added value in their manuscript. They do not present examples of sites of biological interest that could have been missed by conventional data analysis but that were detected only by applying their error correction. Furthermore, the lack of precision in the methods would definitely not allow another research group to reliably use this method in their experiments. This is a major concern which needs to be addressed if the authors want to make their editing efficiency correction approach useful to the rest of the community.

**Reviewer 2**

**Are you able to assess all statistics in the manuscript, including the appropriateness of statistical tests used?** Yes, and I have assessed the statistics in my report.

**Comments to author:**

In the manuscript entitled "Identification of pathogenic variants in cancer genes using base editing screens with editing efficiency correction", Huang et al. developed a method to use base editors to identify pathogenic variants in BRCA1 and BRCA2 genes in a high-throughput manner. They first tested the possibility of base editing screen using 1,908 sgRNAs in four pools (BRCA1-CG-NGG, BRCA2-CG-NGG, BRCA1-AT-NGG, BRCA2-AT-NGG) in eHAP cells and found Ln sgRNA fold changes between day 10 and day 0 could reflect an effect on cell viability. Then, they adapted a high-throughput method to measure the editing outcomes in HEK293T and eHAP cells by pairing sgRNAs and targets in plasmids, and showed that introducing exogenous vectors could reveal the endogenous editing outcomes. In addition, they modeled functional scores of sgRNAs by inferring the relationships between Ln sgRNA fold changes and editing activity. In total, about 416 variants were identified as loss-function mutations for BRCA1/2 by NGG base editors, and 567 variants were identified by NG base editors. Finally, they analyzed the distribution of variants identified in the screen along BRCA1/2 genes and explained some possible mechanisms of pathogenicity of these variants. This work provided a high-throughput strategy to identify pathogenic variants by using base editors; however, some analyses performed in this study seemed preliminary and thus weakened their conclusions. Below please find specific comments for the authors to improve their manuscript.

1. The authors applied two libraries, BRCA1/2-CG-NG and BRCA1/2-CG-NGG, for screen in this study. Although the authors declared that they "correlated well" (Fig. 5C-D), the r < 0.5 is indeed NOT "well" even for the positive controls. In addition, were the same groups of loss-of-function variants identified between two screens? Were most of them overlapped between two screens?

2. The authors selected four newly identified loss-of-function variants for validation and all of them showed effects on delaying cell growth (Fig. 6H). Were they randomly selected or based on some criteria? Were they classified as VUS/Benign/Likely benign or Pathogenic/Likely pathogenic" variants? Were they identified from both screens? It seemed like that the authors wanted to indicate the very high (100%, 4 out of 4) reliability of their finding? The authors might like to test more variants.

3. Only in the discussion, the authors mentioned they assayed 4,864 mutations in BRCA1 and BRCA2 genes. How many of these 4,864 mutations were both selected in two libraries? How did the authors design which of these 4,864 mutations were tested in the BRCA1/2-CG-NG and/or BRCA1/2-CG-NGG libraries?

4. The discussion of this manuscript seemed like not very informative. Many points were needed to be discussed. For example, did the authors think that it is very useful to apply this method for screen variants in other genes than BRCA1 and BRCA2? Were many genes reported to have many cancer-related variants as BRCA1 and BRCA2 and thus worthwhile for this kind of large-scale screen?

5. In Method "Modeling sgRNA functional scores with editing efficiency" part, in 𝛽𝑖 = 𝑙𝑗 (𝑒𝑓𝑖𝑗 − 1) , 𝛽𝑖 is the property for sgRNA i, 𝑒𝑓𝑖𝑗 is the property for sgRNA i in experiment j and 𝑙𝑗 is the property for experiment j. It seemed like that the properties in two sides of the equation were not equal. How did this equation work?

6. In addition to their only method for "Modeling sgRNA functional scores with editing efficiency", previously-reported MAGeCK has been also used in the evaluation of gene screen with CRISPR/Cas in Fig.1 D-G. Did the authors compare these two methods for evaluating sgRNA effects on base editing efficiency? Which one is better and why?

7 In Method "Modeling sgRNA functional scores with editing efficiency" part, "here 0< 𝑝𝑖𝑗 <1, and 𝑝𝑖𝑗(𝑒𝑓𝑖𝑗 − 1) is often small in our experiments, therefore ln(1+ 𝑝𝑖𝑗(𝑒𝑓𝑖𝑗 − 1)) is approximately equal to 𝑝𝑖𝑗(𝑒𝑓𝑖𝑗 − 1)". 𝑝𝑖𝑗(𝑒𝑓𝑖𝑗 − 1) equals to 𝑞𝑖 𝛽𝑖 and dij, and value of dij is range from -0.6 to 0.2 in Fig 3B. Did 𝑝𝑖𝑗(𝑒𝑓𝑖𝑗 − 1) in range from -0.6 to 0.2 still fit to this approximation?

8. It is not clear how functional scores of the positive control in Fig. 4A-D were used to validate the accuracy of this method. Please clarify.

9. Fig. 4F and 5J showed that a large proportion of pathogenic/likely pathogenic variants were not selected to be Loss-of-function variants in this screen, which weakened the effectiveness of this screen. Did the author know why? It may be worthwhile to discuss the disadvantage of this method in this manuscript.

10. eHAP cells and HEK293T cells were both tested in screens. Since eHAP are haploid human cells and HEK293T are triploid, did loss-of-function variants selected by these two types of cells overlap well?

11. It was barely mentioned how to exclude off-target effects in the screen with BEs. Please clarify.

12. Despite the authors found sgRNAs were significantly depleted at day 10 in this study, other reported studies that used CRISPR-Cas9/sgRNA libraries for screening were often transduced about 14 days (PMID: 24336571, PMID: 28933431), 20 days (PMID: 27980086) or even four weeks (PMID: 31727847). The authors may need to re-evaluate their study by sequencing sgRNAs at extended time points, such as day 14 or longer.

**Authors Response**

**Point-by-point responses to the reviewers’ comments:**

Reviewer 1

Major Comments:

1. The manuscript focused on improving the base editing screening method by developing a score model, but the authors did not directly compare the new screening method to the old sgRNA fold-change-based screening methods. I can see that the new method needs more experimental data than the old methods, such as the integrated-genome target library assay. The authors should clearly confirm that the new method has higher screening efficiency or accuracy than the old methods.

This is an excellent suggestion. To compare the performance of our efficiency-corrected fold change score model with other fold-change-based method, we analyzed our data with MAGeCK, the widely used analysis method for identifying essential genes in CRISPR-Cas9 knockout screens. We used all the variants that were annotated as “Pathogenic/Likely pathogenic” in ClinVar database as true positives and those annotated as “Benign/Likely benign” in ClinVar as true negatives. A receiver operating curve (ROC) showed improved sensitivity and specificity for our method in comparison to MAGeCK (AUC=0.66 vs. 0.63). In particular, at a specificity ranging from 0.8 to 0.9, which is a commonly used range for screens, our sensitivity is 1.5-1.8 fold higher than MAGeCK. These results suggested that our method had higher efficiency for identification of pathogenic variants.

We added the comparison in the new Fig. 4g and described the results in the text (page 13-14):

“To evaluate the performance of our efficiency-corrected model on distinguishing true loss-of-function variants from other variants, we calculated the sensitivity and specificity of our classification using “Pathogenic/Likely pathogenic” variants in the ClinVar database as true positives and “Benign/Likely benign” variants in ClinVar as true negatives. Compared to MAGeCK, the area under the receiver operator characteristic curve (ROC-AUC) was higher in our method (0.66 vs. 0.63, **Fig. 4g**). At a specificity ranging from 0.8 to 0.9, our sensitivity is about 1.5 to 1.8-fold higher than MAGeCK (**Fig. 4g**). These results demonstrated our efficiency correction strategy improved the accuracy for identification of loss-of-function variants. ”

2. The authors identified the loss-of-function BRCA1 variants and 530 BRCA2 variants, and validated four sgRNAs using cell viability assays. These four sgRNAs resulted in the delayed growth of eHAP cells compared with non-targeting controls (Fig 6H). However, the control sgRNAs that targeted nonsense mutations should also be tested to evaluate the accuracy of this method. The score of tested sgRNAs should be correlated with their degrees of delayed growth of eHAP.

As suggested, we included the sgRNAs that targeted the nonsense mutations for both *BRCA1* and *BRCA2* genes in the cell growth assay. We used the fold change of absorbance between the tested group and the non-targeting control at day 5 to measure the degree of delayed growth. As the reviewer has predicted, the degree of delayed growth was correlated with the fold change of sgRNA abundance in our screen (Pearson’s r=0.776, p=0.040).

We updated the original Fig. 6H with new data and presented it as the new Fig. 4h and Table S1 in the revised manuscript.

We described the results in the text (paragraph 2, page 14):

“To validate the newly identified loss-of-function variants, we performed cell viability assays on 10 randomly selected variants (Additional file 2: Table S1, **Fig. 4h**). For 7 of the 10 randomly selected LOF sites, treating cells with sgRNAs resulted in the delayed growth of eHAP cells compared with non-targeting controls (Additional file 2: Table S1, **Fig. 4h**). And the level of delayed growth correlated with the degree of sgRNA deletion in the screen (**Fig. 4h**, Pearson’s correlation coefficient r=0.776, p=0.040). ”

3. The complexity of editing outcome is one of the major issues the authors should focus on. In Line 202-203, the authors defined the editing outcome for each sgRNA as the editing result with the highest frequency. The average co-editing frequencies of nucleotides based on their protospacer positions in the editing window of AncBE4max or ABEmax were analyzed in Fig 2 C/D, but the authors did not directly analyze the purity of editing products for each sgRNA. The editing outcome complexity for each sgRNA may still cause perturbations in the model. Moreover, in Line 198, why did the authors analyze "two consecutively Cs or As"? The editing products of AncBE4max and ABEmax base editor are influenced by the chromatin environment, catalytic activity of deamination, editing preference and so on. Analyzing the co-editing frequencies could not reflect that "AncBE4max or ABEmax only produce one type of products".

We thank the reviewer for raising the good question. The complexity of editing outcome was one of the key questions we addressed when identifying loss-of-function variants with base editing screen. Using the integrated genome target site assay, we have directly measured the editing products for each sgRNA on an integrated target DNA region. The different editing products and their relative fractions are now listed in Supplementary Table S5.

Figure 2C/D showed an average of editing results for all the sgRNAs in our library based on their protospacer positions. The purpose of Figure 2C/D and the description of “two consecutively Cs or As” was just to show the complexity of editing outcome, not how we solved the complexity problem. We apologize for not making it clear in the original manuscript.

We solved the complex outcome problem by directly analyzing the editing products for each sgRNA. Our analysis has shown there was a major outcome for most of the sgRNAs. More than 50% of the sgRNAs had only one type of editing product. After merging editing products at the amino acids level, more than 75% of the sgRNAs had a major outcome that was supported by over 80% of the edited reads. Therefore, we defined the major outcome as the variant associated with a sgRNA and used the relative efficiency of generating that specific variant to correct the functional score of the variant in our model. The other outcomes are unlikely to be a major confounding factor for the loss-of-function variant identification.

To clarify the point and better illustrate our solutions for mapping sgRNA effects to variants, we added new Fig. 2c, Fig. 2d and Fig. 2e and the following sentences in the text (paragraph 1, page 10):

“As a result, 41.7% of sgRNAs generated more than one editing products with AncBE4max and 49.7% with ABEmax in the assay (**Fig. 2c,d; Additional file 1:Table S4**). We then merged the editing outcome at amino acid level, i.e. all the edited reads leading to the same amino acid change were counted as the same outcome (**Fig. 2a**). After merging, the fraction of sgRNAs associated with only one or two outcomes increased from 60.2% to 88.8% for AncBE4MAX and from 64.4% to 83.2% for ABEmax (**Fig. 2c,d; Additional file 1:Table S4**). We also found that more than 75% sgRNAs had a major outcome that was supported by over 80% of the editing products (**Fig. 2e**). Therefore, we defined the editing result with the highest frequency as the variant associated with a sgRNA (**Additional file 1:Table S5)**. For each sgRNA, we then calculated the editing efficiency as the fraction of reads with the variant we defined.”

We also agree that the nucleotide sequences were not the only determinant for base editors. Therefore, in our model, we divided the efficiency term into two part, a $q_{i}$ that is determined by sgRNA target sequence, and a $l_{j}$ that is determined by other factors. In our screen, for the same base editor, the catalytic activity of deamination, and editing preference could be treated as the same for all the sgRNAs in the screen library. Therefore, we treated $l_{j}$ as an experiment-specific constant that is incorporated into the functional score $\beta_{ij}$. The comparison of $\beta_{ij}$ for all the variants in the same screen experiment is not affected by the constant. $q_{i}$ is the relative activity of sgRNA $i$ for generating the variant $i$, which was measured from the integrated genome target site assay.

To make it clear, we added the following sentences in the method (page 34):

“Here the percentage of cells with the variant $i$ $p_{ij}$ can be described by

$$p_{ij}=q_{i}l_{j}$$

where $q_{i}$ is the relative activity of sgRNA $i$ for generating the variant $i$, which can be measured separately from our editing efficiency experiment $j$. $l_{j}$ is the efficiency term determined by factors other than sgRNA target site sequence in the experiments, such as the base editor expression level and base editor activities. For the same screen, $l_{j}$ is equal for all the sgRNAs used in the screen. For our analysis, $l_{j}$ is typically not estimated and denotes an experiment-specific constant.”

4. Spearman's correlation coefficient was used to calculate the correlation between the results, which is a method of evaluating the degree of correlation based on ranking data. But this method is not suitable for this study. Other methods should be used, such as Pearson's correlation coefficient. In addition, no matter which method is used, the definition of "good correlations" in this manuscript seems to be too loose. "good correlations" appears many times in the manuscript, but the minimum value of "good correlations" is only 0.466. More statistical methods should be provided to prove the reliability of the results.

Thanks for pointing out the differences in correlation coefficients. As suggested, we now used Pearson's correlation coefficient to evaluate the degree of correlation for all the figures in this work. Also, we changed the statement of "good correlations" to more appropriate expressions throughout the revised manuscript. P-values of all the correlation coefficients have been provided in the figures to evaluate the significance of correlation.

5. In Lines 226-229: the authors stated that "As displayed by simulated data, within conditions of our experiments, we found the natural logarithm (ln) fold change of a sgRNA was almost linearly correlated with the editing activity of the sgRNA". The editing efficiency itself is not high and it likely causes many errors. Besides, it is also found in Fig 2B that it did not conform to a normal distribution or an approximate normal distribution. There was no sufficient evidence to prove this conclusion. It was not correct to use the linear model directly.

We apologize for the misleading description of the model.

The $q_{i}$ in the model does not equal to the fraction of reads edited shown in Figure 2B. As we have shown in the method section and the answers for question #3, the fraction of reads edited with the outcome variant ($p_{ij}$) contain two parts, a $q_{i}$ that is determined by sgRNA target sequence, and a $l_{j}$ that is determined by other factors. We linearly scaled $q_{i}$ to the range of 0.1 to 1, thus the errors in measurement will not cause a big perturbation to the score.

And the linear model in the original manuscript was also misleading. We actually not try to estimate $\beta_{ij}$ by varying $q_{i}$. $q_{i}$ was measured separately from the integrated-genome-target site assay, scaled and used as a constant for each sgRNA. $\beta_{ij}$ was estimated from abundance of sgRNA before and after editing, after correction of $q_{i}$. We now simplified the model to a single formula,

$$\beta_{ij}=d_{ij}/q_{i}$$

Here that functional score $\beta_{ij}$ refers to the most frequent variant generated by sgRNA $i$ in experiment $j$, $d_{ij}$ is the ln fold change of sgRNA $i$ in experiment $j$, $q_{i}$ is the relative editing activity for sgRNA $i$ to generate the most frequent variant (variant $i$). This way the model does not require a normal distribution of $q_{i}$. The linear relationship between $d_{ij}$ and $q_{i}$ is supported by the data of nonsense mutations in Figure 3B and 3C.

We removed the simulated data in original Fig. 3A, modified the text to clarify the model (page 11-12):

“

To precisely determine the functional effects of variants, we proposed a model to correct the effects of sgRNA editing activity in our base editing screen (**Fig. 3a**). For each variant that is the most frequent product after a sgRNA editing, we assume that the observed depletion of the sgRNA is linearly correlated with the editing efficiency for generating the variant. Specifically, for each sgRNA $i$ in the screen experiment $j$, the functional score for the most frequent variant generated by sgRNA $i$ (variant $i$) in experiment $j$, $\beta_{ij}$, can be modeled as:

$$\beta_{ij}={d_{ij}/q}_{i}$$

here$d_{ij}$ is the ln fold change of sgRNA $i$ in experiment $j$, relative to the median ln fold change of negative control sgRNAs in that experiment. $q_{i}$ is the relative editing activity for sgRNA $i$ to generate variant $i$, which can be measured experimentally or predicted computationally. We also showed that the linear relationship could be approximated from a simple exponential cell growth model (See Methods, “Modeling sgRNA functional scores with editing efficiency”).

Consistent with the model, for the sgRNAs that could generate premature stop codons in *BRCA1*, which may be equivalent to a group of nonsense variants that having the same $\beta$ score, we observed an inverse linear relationship between ln fold change and the relative efficiency of nonsense variant production (**Fig. 3b**, Pearson’s r=-0.507, p=0.019). Similar trend was also observed for sgRNAs that could generate nonsense variants in *BRCA2* (**Fig. 3c,** Pearson’s r=-0.554, p=0.003).”

We also added in the discussion, that the linear model may not apply to all BE screens (paragraph 1, page 20):

“It is noted that although efficiency-correction is needed for better identification of loss-of-function variants in all BE screen experiments, the linear relationship between ln fold change and editing efficiency may not directly apply for other base editing screens. Other efficiency-correction models may be developed under our framework to improve variant classification.”

6. The results of Fig4 E/F (NGG) and Fig5 E/F (NG) were quite different, especially in the "Stop gain" group. The authors have to clarify it. Is it because of the different library content of NG and NGG?

Yes, the library content of NG and NGG are different. The two libraries had only a small fraction of shared targeted variants, including 22 stop gains.

We think the lower rate of “stop gain” mutation identified as “loss-of-function” in the NG library is likely due to the lower editing efficiencies of “NG” recognizing base editors in comparison to “NGG” recognizing base editors. When the percentage of edited cells were lower than a threshold, it would not generate a robust signal in the screen. Supporting this hypothesis, we found that filtering out sgRNAs with low editing efficiencies increased the estimated sensitivity (i.e., the percentage of pathogenic identified as LOF variants in the screen) from 46.9% to 51.2% in our NGG screen.

We added in the text to explain the difference (page 14):

“Filtering out the sgRNAs that had lower editing activity further increased the sensitivity from 46.9% to 51.2% in our model (**Fig. 4g**), suggesting that increasing editing efficiency of base editing may improve the sensitivity of the screen. “

And on page 15:

“The sensitivity for identifying “Pathogenic/Likely pathogenic” variants was 2.2 fold lower for screens with NG base editors compared to screens with NGG base editors, likely due to the low efficiency of NG base editors.“

Minor Comments:

1.	Line 111-113: "In a pooled screen, the change of sgRNA abundance between two conditions is often used to evaluate the effects of sgRNAs". Related reference should be cited here.

We have cited the following reference:

Hanna, R.E. and J.G. Doench, Design and analysis of CRISPR–Cas experiments. Nature Biotechnology, 2020.

2.	Line 141-158: what are the reasons for choosing these three sgRNAs? Why not repeat the same test in BRCA2 gene region? The sequences and other detailed information of selected three sgRNA are missing.

We chose the three sgRNAs to validate that base editors AncBE4max and ABEmax could generate variants in eHAP cells. The three sgRNAs are expected to generate two known loss-of-functional variants and one neutral variant of *BRCA1* after base editing. A similar test was not performed for sgRNAs targeting *BRCA2* because we think the data is sufficient to prove the feasibility of base editing in eHAP cells. We have added the sequences for the selected sgRNAs in Table S9.

3.	Line 159: "1,908 sgRNAs", only 728 sgRNAs were found in Table S1.

We apologize for missing these sgRNAs in Table S1. We now attached the correct Table S1 (now named Table S2) with all the sgRNA sequences used in the NGG and NG screens, and marked the non-targeting control sgRNAs, positive control sgRNAs and the 47 sgRNAs targeting SINE/Alu.

4.	Line 162-163: Related sequences or other detailed information about non-targeting sgRNAs are missing.

Please see the answer to question #3.

5. Line 176-177: Related sequences or other detailed information about 47 sgRNAs targeting SINE/Alu are missing.

Please see the answer to question #3.

6. Line 177: Please explain how to determine SINE/Alu repeats and cite related references.

We retrieved the information about the SINE/Alu repeats in UTR regions of *BRCA1/2* from UTRdb (<http://utrdb.ba.itb.cnr.it/>). The following reference was added:

Grillo, G., et al., *UTRdb and UTRsite (RELEASE 2010): a collection of sequences and regulatory motifs of the untranslated regions of eukaryotic mRNAs.* Nucleic Acids Res, 2010. **38**(Database issue): p. D75-80.

7. Line 191-193: The explanation of orange dash is missing in figure legend of Fig. 2B. Is it average or median?

The orange line represents median of the fraction of edited reads for all sgRNAs in the library. We have added this information in the figure legend in the revised manuscript.

8. Line 270-274: "These results suggested the assay was able to provide functional assessment for variants in both coding and noncoding regions of genes". Please clarify it.

We performed base editing screens across all exon regions in *BRCA1* and *BRCA2*. Here the noncoding regions referred to the 5’- and 3’-untranslated regions and the “coding regions” referred to the translated regions. We think the sentence did not provide useful information and removed the it to accommodate changes based on other suggestions.

9. Line 468: "a 82 bp protospacer" should be "an 82 bp sgRNA Scaffold".

Thanks for pointing it out. We have corrected it.

10. Fig 1B: In Fig 1B, different categories are difficult to distinguish; same problem in Fig S1. More colors could be used.

Thanks for the suggestion, we now use different colors to represent different categories in the growth curves in Fig. 1B and Fig. S1.

11.	Table S1: What is the meaning of "C-G_NGG" in lib_type? it looks like GBE (C:G to G:C conversion) library.

Sorry for the misleading annotation. Here C-G means targeting sites are cytosines and guanines rather than “C:G to G:C transversion”. As described in line 159-162, “1,908 sgRNAs targeting BRCA1/2 were designed divided into four pools, named BRCA1-CG-NGG and BRCA2-CG-NGG for cytosine base editing, and BRCA1-AT-NGG and BRCA2-AT-NGG for adenine base editing”.

For clarity, we changed "C-G_NGG" to "BRCA1-CG-NGG" or “BRCA2-CG-NGG” in the updated Table S1 (now named Table S2).

12.	The significance test method used in this study should be given.

We added statistical test information and P-values in the revised manuscript wherever applicable.

13.	Many details about the positive and negative controls are missing in this manuscript and some figures could be moved to Supplementary Materials.

Thank you for the suggestion. We added information about positive and negative controls in the manuscript and detailed information in Supplementary tables. We also reorganized the figures, deleted the original Fig. 3A, Fig. 5C, and Fig. 5D, and moved figure 3C and 3D to Supplementary Materials.

Reviewer #2: In "Identification of pathogenic variants in cancer genes using base editing screens with efficiency correction", Huang & Li et al leverage base editors to investigate the effect of thousands of mutations in the BRCA1 and BRCA2 genes. They perform 8 different screens (2 BEs x 2 genes x 2 Cas9) to enhance the number of variants characterized, while also performing large-scale editing efficiency testing of their gRNAs in parallel. They then use this data to develop a model that adjusts the essentiality scores to take into account the intrinsic efficiency of each gRNA.

Overall, I found the manuscript to be well written and interesting. I particularly enjoyed the idea of performing fitness assays in parallel with editing efficiency assays, which has the potential to address an important bias in large-scale base editing applications. I think the content of the manuscript is of interest to many in the genome editing community as well as those interested in genotype-to-phenotype maps. That being said, I think the manuscript needs much improvement before it is ready for publication. In particular, I found a lot of data to be missing and that multiple aspects of the method could be better explained. Here are my comments:

1) There have been multiple studies that used recent advances in genome editing to characterize the effect of BRCA1 variants, including one that used the BE3 base editor in HAP1 cells. While the authors cite these studies (Findlay et al 2018 and Kweon et al 2020), they should also compare their results and discuss whether or not there is a significant overlap in the loss of function (LOF) mutants they observe. Furthemore, the authors should cite Findlay et al 2018 as well Blomen et al 2015 (Science) when discussing the cell growth phenotypes of LOF mutants, as these genes had already been shown to be essential in Haploid human cells.

We appreciate the reviewer’s suggestion. We have compared our results with the two published studies on the variant effects of *BRCA1*. 11 LOF variants we identified were also classified as LOF in Findlay et al., and 9 of them were also identified as LOF in Kweon et al.. The overlap of loss-of-function variants were significant between our study with both of the two studies (Fisher's exact test pvalue:0.004 and 0.00008, respectively).

We added the results in the text (paragraph 2, page 13)

“Our loss-of-function variants overlapped significantly with previously reports **(Additional file 2: Table S8,** Fisher's exact test, p<0.005). 11 of our *BRCA1* LOF variants were also classified as deleterious variants in a saturated genome editing [11]. And we identified 9 of the 19 LOF variants in a base editing screen targeting *BRCA1* [21].”

We added the two references to the sentence at line 138-140:

“Reducing the expression levels of *BRCA1* or *BRCA2* by RNAi in eHAP cells inhibited the cell growth (**Additional file 1: Fig. S1a-d**), confirming that these two genes are essential in haploid human cells [11,27]. ”

2) In multiple parts of the manuscript, there is not enough information on the statistical tests or observations performed. For example, no p-values are provided for any of the correlations presented in the main or supplementary figures.

We thank the reviewer for pointing out the problem. We have added detailed information on statistical tests including p-values throughout the manuscript.

3) The authors should discuss why there seems to be an important difference in replicate correlation between the AT and CG screens (as shown in figure S2)

We think the difference in replication correlation is related to differences in the signal levels between AT and CG screens. When majority of the sgRNA only had minor effects on cell growth, we would expect to see low correlation between replicates. Only the C->T conversion could generate stop gain variants, which could lead to complete loss of function of *BRCA1/2*. Also, we have put positive control sgRNAs that targeting a group of essential genes other than *BRCA1/2* in CG screens. Therefore, we would expect that CG screens had more signals and thus higher replicate correlation than AT screens.

In fact, in the figures presented in our original manuscript, the sgRNAs with off-target sites (sgRNAs targeting more than three sites in the genome) were removed from presentation. Because many of the off-target sgRNAs had strong signals, after adding back these sites, the overall correlations became better and the differences between AT and CG screens were not significant.

We updated the Fig. S2a-d to include all the sgRNAs used in the screen. Those sgRNAs with off-target sites were labeled as red dots.

4) Based on the information provided in the text and in Figure 2, I worry that the sequencing coverage used in the genomic integration BE assay is too low to accurately measure which editing outcomes are the most frequent. As stated on line 192, the median target site coverage for each gRNA was 4000 reads. If we consider a median editing rate of 4.6% (as stated on line 193), this would mean that the "editing activity" was often calculated based on very few reads (e.g. : a gRNA with an editing rate of 2% and a coverage of 2000 reads = 40 reads). It might be useful to add minimal depth thresholds for editing "editing activity" determination so that only gRNAs with sufficient coverage are kept. This bias would also affect the co-editing efficiency analysis. I saw that there is a 30 reads or 0.005 frequency cut-off in the "editing efficiency correction" section (703-704), but it is unclear how this is taken into account in the rest of the manuscript.

Yes, we used both the 0.005 frequency and 30 reads minimal depth as cutoff and applied it for all the editing efficiency analysis. In fact, after setting the frequency of 0.005, only about 50 (0.9%) sgRNAs had a coverage of lower than 100 reads. The coverage of our genomic integrated target site library assay was in agreement with other studies of base editing efficiency[1], in which they used 100 reads as a minimal depth cutoff but did not use the frequency cutoff.

We added the threshold information in the “**Editing outcome and efficiency mapping using data from integrated-genome target library assay”** (paragraph 2, page 33)**:** “sgRNAs having editing outcome with coverage less than 30 reads or frequency below 0.005 in both replicates of the genome-integrated target site assay were removed for further analysis.”

5) Related to this issue, there is not enough information available on the integrated target genome site library assay for the NG editor libraries. Was the median coverage identical to the NGG editor libraries? How well did the different replicates correlate?

Yes, the median coverage of reads in the integrated target genome site library assay for the NG base editor was also over 4000, identical to that of the NGG base editor. For AncBEmax_NG and ABEmax_NG, the Pearson’s correlation efficiency between replicates were 0.912 and 0.823, respectively. We added new Figure S3e and S3f showing the correlation between two replicates of NG screens.

6) I have some interrogations on the data presented in figure 2 E and F. First, both axes of both panels should be scaled identically, and in a more intuitive way (e.g.: higher editing rates = higher value on the axis). Second, If I understood correctly lines 587-588 in the methods, gRNAs for which no editing could be observed were excluded from this figure and the correlation coefficient calculations? If so, could the authors justify this omission? It seems like important information to know that a subset of gRNAs did not work at all in the eHAP cell lines.

We thank the reviewer for comments #6, #7, #8 and #9, which pointed out the issues in Figure 2,3,4 and 5 to help us improve the presentation of data. All these figures have been updated as suggested.

For endogenous editing efficiency mapping, we omitted the gRNAs that did not have more mutations than the control samples (no base editor transfected) in targeted amplicon sequencing. We interpreted these gRNAs as those had low editing efficiencies that could not be reliably detected in amplicon sequencing due to the errors in PCR and sequencing. The purpose of Figure 2E and 2F was to show that the editing efficiency measured by integrated genome target site was in agreement with the endogenous editing efficiency. The omission of these sgRNAs did not affect the conclusion.

7) The same comment applies to panels B and C of figure 3. The y axis should have the same scaling.

The figure has been updated as suggested.

8) The same comment applies to Figure 4 panels A, B, C and D. The y-axis should be scaled based on density, not absolute numbers, and the histogram bin size should be the across all categories and panels.

The figure has been updated with density on y-axis, and the same bin size across all the panels on x-axis. We removed the distribution of negative controls in the figure because it did not provide useful information other than showing that the scores for non-targeting sgRNAs followed an approximately normal distribution.

9) Finally, this comment also applies to figure 5 C, D and E, F, G, H. The axis for the scatter plots shown in figure 5 C and D should be scaled the same way. For panels E, F, G, H, the y-axis should be scaled based on density, not absolute numbers, and the histogram bin size should be the across all categories and panels.

The figure has been updated as suggested.

10) The authors should provide the beta score data for the NG editor screens, not just for the NGG editor experiments.

We now included the information, including the MAGeCK beta score, p-values and fdr for NG editors in Table S2.

11) The methods section on the Gaussian mixture modeling is severely lacking in detail. While this can be partially addressed by making the computer code that underlie it open access, there should more information on how the data was processed. The authors should provide statistical support for their 4 nuclei model and provide the AIC values associated with the different hypothesis they tested.

We thank the reviewer for the good suggestion. The differences of AIC or BIC values are not significant when the nuclear parameters are from 2 to 6. Our assumption of the data were that most variants have no impact on the function, which should presented by a null distribution. Also, for better classification, we wanted to minimize the overlap of the distribution for loss-of-function variants with the distribution of null distribution. When we look at the model with 2, 3 and 4 Gussian components, we found that a mixture model of four Gaussian components best fitted the our assumption and expectation. Therefore, a mixture model with four Gaussian components were used to fit the distribution of the score.

We now provided the AIC and BIC table in Table S7, the fitted distributions with 2,3 and 4 nuclei in Fig. S5 and provided the information about our choice in the text (paragraph 2, page 12):

“By fitting the score distribution with a Guassian mixture model, we found that models with 2 to 6 nuclei had small differences in AIC/BIC values (**Additional file 1: Table S7)**. We chose the model of four Gaussian components because they best fitted our assumptions of the data (**Additional file 1: Fig. S5a-l**).”

We also provided the code of the analysis.

12) As this manuscript contains a lot of computational work and presents a new method for large-scale BE scree data analysis, all the codes used should be made available. This includes both the code used to perform the data analysis and to generate the figures as well as the sequencing analysis pipelines.

We have provided all the codes we used for performing the data analysis and generating all the figures. It is now available at https://github.com/guangyu1990/Edit_Efficiency_Correction.

13) I think the manuscript would be much improved by additional validation experiments and follow up on screen hits. Furthermore, I think the validation experiments would be stronger if the mutant cell lines were not generated by BE but by other another editing approach. Having some mutant specific assays (such as expression measurements for the UTR mutations) would also be a plus.

We thank the reviewer for the good suggestion. We have performed more validation experiments. While it was difficult to generate a mutant cell line using another editing approach, we have found that mRNA levels of *BRCA1/2* decreased by introducing specific C to T mutations at UTR regions using base editors. The new data were included in the new Figure 6h. We also added more validation experiments in the new Figure 4h.

We described the results in the text (paragraph 2, page 17):

“For 6 sgRNAs that inhibited the growth of eHAP cells in a cell viability assay (**Additional file 1: Fig. S7**), we found that four of them reduced the mRNA levels of *BRCA1* or *BRCA2* (**Fig. 6h**), suggesting that their associated variants had direct impacts on *BRCA1/2* function through regulation at mRNA level.”

14) Finally, I think that while the editing efficiency correction idea is splendid, the authors do not show its added value in their manuscript. They do not present examples of sites of biological interest that could have been missed by conventional data analysis but that were detected only by applying their error correction. Furthermore, the lack of precision in the methods would definitely not allow another research group to reliably use this method in their experiments. This is a major concern which needs to be addressed if the authors want to make their editing efficiency correction approach useful to the rest of the community.

We thank the Reviewer for the good suggestion, indeed, many loss-of-function variants will be missed if editing efficiency was not corrected. We analyzed our data with MAGeCK, the widely used analysis method for identifying essential genes in CRISPR-Cas9 knockout screens. We found that at the p-value less than 0.05, only 50 variants were considered loss-of-function by MAGeCK. Among them, only 1 is annotated as “Pathogenic/likely pathogenic” in Clinvar.

We then compared the performance our method with MAGeCK using all the variants that were annotated as “Pathogenic/Likely pathogenic” in ClinVar database as true positives and those annotated as “Benign/Likely benign” in ClinVar as true negatives.

The area under the receiver operator characteristic curve (ROC-AUC) was higher in our method (0.66 vs. 0.63). In particular, at a specificity ranging from 0.8 to 0.9, our sensitivity is 1.5-1.8 fold higher than MAGeCK. These results suggested that our method significantly improved the identification of loss-of-function variants using BE screens. We added the comparison in new Figure 4g and described the result in the text (page 13-14).

We also added more detailed information about our method in the text, figure legend as well as supplementary tables to make the method easy to follow. In the discussion, we discussed possible strategies methods to improve the precision and base editing efficiency in the future.

Reviewer #3: In the manuscript entitled "Identification of pathogenic variants in cancer genes using base editing screens with editing efficiency correction", Huang et al. developed a method to use base editors to identify pathogenic variants in BRCA1 and BRCA2 genes in a high-throughput manner. They first tested the possibility of base editing screen using 1,908 sgRNAs in four pools (BRCA1-CG-NGG, BRCA2-CG-NGG, BRCA1-AT-NGG, BRCA2-AT-NGG) in eHAP cells and found Ln sgRNA fold changes between day 10 and day 0 could reflect an effect on cell viability. Then, they adapted a high-throughput method to measure the editing outcomes in HEK293T and eHAP cells by pairing sgRNAs and targets in plasmids, and showed that introducing exogenous vectors could reveal the endogenous editing outcomes. In addition, they modeled functional scores of sgRNAs by inferring the relationships between Ln sgRNA fold changes and editing activity. In total, about 416 variants were identified as

loss-function mutations for BRCA1/2 by NGG base editors, and 567 variants were identified by NG base editors. Finally, they analyzed the distribution of variants identified in the screen along BRCA1/2 genes and explained some possible mechanisms of pathogenicity of these variants. This work provided a high-throughput strategy to identify pathogenic variants by using base editors; however, some analyses performed in this study seemed preliminary and thus weakened their conclusions. Below please find specific comments for the authors to improve their manuscript.

1. The authors applied two libraries, BRCA1/2-CG-NG and BRCA1/2-CG-NGG, for screen in this study. Although the authors declared that they "correlated well" (Fig. 5C-D), the r < 0.5 is indeed NOT "well" even for the positive controls. In addition, were the same groups of loss-of-function variants identified between two screens? Were most of them overlapped between two screens?

We apologize for the misleading presentation in Fig.5C and 5D, in fact, those “positive controls” are not indicators of reliability of our assay. First, those positive controls are not sgRNAs targeting *BRCA1/2*, but a group of essential genes. These genes had different degrees of essentiality in HAP1 cells. Second, due to the availability of PAM sequences and our design rules (see Methods “Oligonucleotide-library design and synthesis”), the the sgRNA sequences for the same positive control genes are always different for the two libraries. As a result, even for the same positive control variant, its fraction in the final editing products are different in the two screens. Therefore,the positive controls in two libraries should not be treated as replicates. Their correlation level is not a direct evidence of reliability of our screen. We tried to use the original Fig. 5C-D to show that the functional scores of positive controls in the BRCA1/2-CG-NG screen was lower than the scores in the BRCA1/2-CG-NGG library. We think it is due to the lower editing efficiencies of NG base editors than the NGG base editors. Because these figures did not provide any evidence to support or against our conclusion, to avoid confusion, we now deleted the original Figure 5C-D.

For the second question, we actually used four sets of libraries, *BRCA1/2-CG-NGG*, *BRCA1/2-AT-NGG*, *BRCA1/2-CG-NG* and *BRCA1/2-AT-NG*. The first two libraries were screened with NGG recognizing base editors and the later two libraries were screened by NG-recognizing base editors. There were 729 shared variants between screens using NGG recognizing base editors and screens using NG-recognizing base editors. Over 74% of them were concordant in functional classification, including 35 loss-of-function variants. The overlap of loss-of-function variants between the two screens was significant (Fisher’s exact test, p=0.001). We added the information in the text (paragraph 1, page 15 ):

“Among the 729 variants that were shared between screens using “NGG” recognizing base editors and screens using “NGN” recognizing base editors, over 74% of them were concordant in functional classification, including 35 loss-of-function variants (67 LOF variant only in NG and 125 LOF variant only in NGG, Fisher’s exact test, p=0.001). “

2. The authors selected four newly identified loss-of-function variants for validation and all of them showed effects on delaying cell growth (Fig. 6H). Were they randomly selected or based on some criteria? Were they classified as VUS/Benign/Likely benign or Pathogenic/Likely pathogenic" variants? Were they identified from both screens? It seemed like that the authors wanted to indicate the very high (100%, 4 out of 4) reliability of their finding? The authors might like to test more variants.

We randomly selected these four sites for validation. As suggested, we tested 6 more variants. Together, 7 of the 10 variants were validated to be able to inhibit cell growth. And the level of delayed growth were correlated with the degree of sgRNA depletion in the screen. These results are in agreement with the 40-50% accuracy we estimated for our screen based on the pathogenic/Likely and benign/likely benign mutations in Clinvar. The new data is presented in the new Fig. 4h and Table S1.

We described the results in the text (paragraph, page 14):

” To validate the newly identified loss-of-function variants, we performed cell viability assays on 10 randomly selected variants (Additional file 2: Table S1, **Fig. 4h**). For 7 of the 10 randomly selected LOF sites, treating cells with sgRNAs resulted in the delayed growth of eHAP cells compared with non-targeting controls (Additional file 2: Table S1, **Fig. 4h**). And the level of delayed growth correlated with the degree of sgRNA deletion in the screen (**Fig. 4h**, Pearson’s correlation coefficient r=0.776, p=0.040).”

3. Only in the discussion, the authors mentioned they assayed 4,864 mutations in BRCA1 and BRCA2 genes. How many of these 4,864 mutations were both selected in two libraries? How did the authors design which of these 4,864 mutations were tested in the BRCA1/2-CG-NG and/or BRCA1/2-CG-NGG libraries?

We designed the target sites for editing based on the availability of PAM sequences for sgRNAs. For all *BRCA1/2* exons, we searched for targetable sites (C or A) within the 13-17bp window upstream of an NGG or NG PAM sequence. As we have stated in the text, for the NGG screen “1,908 sgRNAs targeting *BRCA1/2* were designed and divided in four pools, named *BRCA1*-CG-NGG and *BRCA2*-CG-NGG for cytosine base editing, and *BRCA1*-AT-NGG and *BRCA2*-AT-NGG for adenine base editing”. For the NG screen: “Having fully established the assay, we next applied the method to assess 7,450 bases in *BRCA1/*2”.

Because in base editing a sgRNA could modify more than one nucleotide in the activity window for one site, the variant representing the major outcome of sgRNA editing is only available after outcome mapping. That is why we showed the mutation number only after outcome and efficiency mapping using integrated genome-target site assay. The 4,864 mutations are the total number of mapped variants from four sets of screens, namely *BRCA1/2-CG-NGG*, *BRCA1/2-AT-NGG,* *BRCA1/2-CG-NG* and *BRCA1/2-AT-NG*. As we mentioned in the response to question #1, after mapping, we found that there were 729 shared variants between screens of NGG base editors and screens with NG base editors.

For clarity, we now added the library information for the mapped variants in Table S6. The shared variants were also marked in the table.

4. The discussion of this manuscript seemed like not very informative. Many points were needed to be discussed. For example, did the authors think that it is very useful to apply this method for screen variants in other genes than BRCA1 and BRCA2? Were many genes reported to have many cancer-related variants as BRCA1 and BRCA2 and thus worthwhile for this kind of large-scale screen?

Thanks for the suggestion. Other cancer risk genes also had many cancer-related variants reported in the ClinVar database. For example, there are 5,842 variants for the *ATM* gene alone. We discussed how our approach could be used to study these variants in the future (paragraph 3, page 19):

“Our efficiency-correction framework is readily applied to identification of LOF variants in other cancer genes. Genes that are essential in eHAP cells include some cancer risk genes such as *PALB2*, *BARD1*, *RAD51C and RAD51D*, which have thousands of variants recorded in ClinVar [2]. About two-third of the variants in these genes could be directly assessed using the same approach presented in this study. For genes that are not essential in eHAP cells, base editing screens with the efficiency-correction is still applicable if a proper functional assay is developed to enrich or deplete the LOF variants.”

5. In Method "Modeling sgRNA functional scores with editing efficiency" part, in $\beta_{i}={l_{j}(e}^{f_{ij}}-1)$ , $\beta_{i}$ is the property for sgRNA i, $f_{ij}$ is the property for sgRNA i in experiment j and $l_{j}$ is the property for experiment j. It seemed like that the properties in two sides of the equation were not equal. How did this equation work?

We thank the reviewer for pointing out the annotation error in the equation. Indeed here $\beta_{i}$ should be $\beta_{ij}$, the functional score for the most frequent variant generated by sgRNA $i$ (variant $i$) in experiment $j$. We revised the modeling part in method to reflect the changes (page 34-35):.

“Then we define the functional score $\beta_{ij}$ for variant $i$ in experiment $j$ as $\beta_{ij}={l_{j}(e}^{f_{ij}}-1)$. We have the following formula for calculating the functional score $\beta_{ij}:$

$\beta_{ij}={d_{ij}/q}_{i}$(4)	”

Also, we clarified $l_{j}$ (page 34):

”$l_{j}$ is the efficiency term determined by factors other than sgRNA target site sequence in the experiments, such as the base editor expression level and base editor activities. For the same screen, $l_{j}$ is equal for all the sgRNAs used in the screen. For our analysis, $l_{j}$ is typically not estimated and denotes an experiment-specific constant. “

6. In addition to their only method for "Modeling sgRNA functional scores with editing efficiency", previously-reported MAGeCK has been also used in the evaluation of gene screen with CRISPR/Cas in Fig.1 D-G. Did the authors compare these two methods for evaluating sgRNA effects on base editing efficiency? Which one is better and why?

As suggested, we used MAGeCK-MLE to analyze our base editing screen data with sgRNA guide efficiency considered. The MAGeCK-MLE algorithm identifies essential genes from CRISPR-knockout screens while considering the sgRNA knockout efficiencies by a maximum likelihood estimation. The algorithm starts with a 100% efficiency for all sgRNAs if no initial guess was made. As shown in the Figure below, if we do not supply initial estimates of sgRNA efficiencies, MAGeCK gave all the efficiency values as 1. After we supplied an initial estimate for sgRNA efficiencies, all the efficiency values given by MAGeCK were still close or equal to 1, which is clearly not the case for base editing screens.

The poor performance of MAGeCK on evaluating sgRNA effects on base editing efficiencies can be explained by the design of MAGeCK. MAGeCK is designed for analyzing CRISPR knockout screens that multiple sgRNAs targeting one gene at relatively high efficiencies. MAGeCK-MLE combines information from multiple sgRNAs targeting the same gene to estimate their efficiencies and the gene essentiality. Our base editing screen is equivalent to the case that only one sgRNA is available for a gene in the CRISPR knockout screen and the efficiencies are variable among sgRNAs. Therefore, the maximum likelihood method in MAGeCK is not possible to accurately predict the base editing efficiency.

Nevertheless, a direct comparison of our method with MAGeCK-MLE on sgRNA efficiency estimation is not applicable. Unlike MAGeCK, we actually experimentally measured the sgRNA efficiencies through a high throughput integrated genome target site library assay. Our model takes the measured efficiencies to correct the functional scores of variants for identification of loss-of-function variants.

Therefore, we compared our method with MAGeCK on the performance on identification of loss-of-function variants. Using all the variants that were annotated as “Pathogenic/Likely pathogenic” in ClinVar database as true positives and those annotated as “Benign/Likely benign” in ClinVar as true negatives, we found that our method had higher sensitivity and specificity than MAGeCK.

We now added a new figure 4g to show the comparison of our method with MAGeCK.

7 In Method "Modeling sgRNA functional scores with editing efficiency" part, "here 0<$p_{ij}$ <1, and $p_{ij}(e^{f_{ij}}-1)$ is often small in our experiments, therefore ${ln(1+p}_{ij}(e^{f_{ij}}-1))$ is approximately equal to $p_{ij}\left( e^{f_{ij}}-1 \right)$". ${ln(1+p}_{ij}(e^{f_{ij}}-1))$ equals to ${ln(C}_{ij}/S_{ij})-\hat{b_{j}}$ and dij, and value of dij is range from -0.6 to 0.2 in Fig 3B. Did$p_{ij}\left( e^{f_{ij}}-1 \right)$in range from -0.6 to 0.2 still fit to this approximation?

We apologize for the confusing presentation. In our work, we actually proposed the linear model first and then found that the linear relationship is suffice to describe our data in Fig. 3B-3C. We then tried to justify the linear model by derivation it from an exponential cell growth assumption in the method. Indeed the approximation may not be perfect theoretically, even though our data in Fig. 3B is largely consistent with the linear relationship and not sensitive to departure from the approximation. To clarify the point, we modified the text and figures to reflect our reasoning process (page 11 to 12).

“To precisely determine the functional effects of variants, we proposed a model to correct the effects of sgRNA editing activity in our base editing screen (**Fig. 3a**). For each variant that is the most frequent product after a sgRNA editing, we assume that the observed depletion of the sgRNA is linearly correlated with the editing efficiency for generating the variant. Specifically, for each sgRNA $i$ in the screen experiment $j$, the functional score for the most frequent variant generated by sgRNA $i$ (variant $i$) in experiment $j$, $\beta_{ij}$, can be modeled as:

$$\beta_{ij}={d_{ij}/q}_{i}$$

here$d_{ij}$ is the ln fold change of sgRNA $i$ in experiment $j$, relative to the median ln fold change of negative control sgRNAs in that experiment. $q_{i}$ is the relative editing activity for sgRNA $i$ to generate variant $i$, which can be measured experimentally or predicted computationally. We also showed that the linear relationship could be approximated from a simple exponential cell growth model (See Methods, “Modeling sgRNA functional scores with editing efficiency”).

Consistent with the model, for the sgRNAs that could generate premature stop codons in *BRCA1*, which may be equivalent to a group of nonsense variants that having the same $\beta$ score, we observed an inverse linear relationship between ln fold change and the relative efficiency of nonsense variant production (**Fig. 3b**, Pearson’s r=-0.507, p=0.019). Similar trend was also observed for sgRNAs that could generate nonsense variants in *BRCA2* (**Fig. 3c,** Pearson’s r=-0.554, p=0.003). ”

8. It is not clear how functional scores of the positive control in Fig. 4A-D were used to validate the accuracy of this method. Please clarify.

We did not present functional scores of the positive control in Fig. 4A-4D. We apologize for the confusing presentation in the figures. And here is the information we would like to clarify about positive and negative controls in our screen. The positive controls we mentioned in the text were 230 sgRNAs targeting 88 essential genes other than *BRCA1/2*. These genes were selected from the fitness gene list of HAP1 cells [3], they may have different degrees of essentiality in eHAP. The negative controls were a group of non-targeting sgRNAs. We presented functional scores of negative control sgRNAs as orange bars in Fig. 4A-4D just to show that they followed a normal distribution as expected. We have compared the positive and negative controls in Additional file 1: Fig. S2E-F, which indicated positive controls were more likely to be depleted than negative control sgRNAs.

For clarity, we removed the scores of negative controls in Fig. 4a-d. We also added the detailed information on the positive and negative sgRNAs in Table S2.

9. Fig. 4F and 5J showed that a large proportion of pathogenic/likely pathogenic variants were not selected to be Loss-of-function variants in this screen, which weakened the effectiveness of this screen. Did the author know why? It may be worthwhile to discuss the disadvantage of this method in this manuscript.

We think several limitations in the base editors may affect the sensitivity of LOF variants detection. First, most variants could only be generated by one sgRNA using base editors. Thus, our base editing screen is equivalent to a CRISPR KO or a CRISPR interference screen for essential genes, with only one sgRNA designed for a gene. It has been shown in Daley et al., for CRISPRi screens, it is impossible to correlectly identify all of the essential genes with a low number of sgRNAs targeting one gene [4]. In fact, when only one sgRNA per gene is used, they estimated the sensitivity to be around 0.2, i.e. only 20% true positives could be identified in CRISPRi screens.

In our case, in Fig. 4F, with efficiency-correction, we identified 47% of pathogenical/likely pathogenic variants in the screen with “NGG” recognizing base editors. We think variants generated by sgRNAs with lower editing activity are more likely to be missed. As shown in Fig. 3B, when the relative editing activity of the sgRNA is lower than 0.2, most of the ln fold change is between -0.2 to 0.2, which is a low signal in the screen that could not be measured precisely.

In Figure 5J, we identified 21% of the pathogenic/likely pathogenic variants. The lower sensitivity of identifying pathogenic variants in Figure 5J was likely due to the lower efficiency of the “NG” recognizing base editors in comparison to “NGG” recognizing BE4max and ABEmax. The base editor efficiency was the major limitation of our screen that may be improved by using new versions of base editors in the future.

We have discussed the major limitations of the methods and suggested ways to improve the screen in the future (paragraph 2, page 20):

“Although our approach has addressed the problem of variability in guide RNA editing efficiency, several problems with current versions of base editors set the limitations for functional assessment of variants using BE screens. First, the targeting scopes of base editors are constrained. Only four types of transition point mutations are available for current CBE or ABE base editors. And current versions of base editors based on Cas proteins still require a PAM sequence, which further limits the number of sites that could be edited [5]. Second, base editors can modify more than one nucleotide in the editing activity window, preventing the assessment of some single nucleotide variants. Moreover, the low editing efficiency for some of the base editors reduced the sensitivity for identifying known pathogenic variants, as we have observed for the “NGN” recognizing base editors in comparison to “NGG” recognizing base editors. Therefore, base editors with broader targeting scope, more precision and higher editing efficiencies are needed. ”

10. eHAP cells and HEK293T cells were both tested in screens. Since eHAP are haploid human cells and HEK293T are triploid, did loss-of-function variants selected by these two types of cells overlap well?

We apologize for the confusion. We did not use the HEK293T cells for base editing screen. The HEK293T were used to perform the genome integrated target site assay to measure the efficiency of base editing. We have shown in Figure 2E and 2F that the editing efficiency measured in HEK293T cells were correlated with the endogenous editing efficiencies in eHAP cells.

11. It was barely mentioned how to exclude off-target effects in the screen with BEs. Please clarify.

For sgRNA dependent off-target effects, we have excluded the sgRNAs with more than three target sites from the final analysis, as described in the last part of “Base editing screens for loss-of-function variants in BRCA1 and BRCA2 in eHAP human haploid cells” in the manuscript.

For sgRNA independent off-target effects, such as those introduced by base editors, we treated them as random noises. As shown by Shuai Jin et al, BE induced mutations were distributed throughout the genome without hotspots [6]. In a population of hundreds of cells infected with a specific sgRNA, for each variant generated by off-target effects of BE, its fraction will be tens to hundreds fold smaller comparing to the main outcome generated by on-target base editing of the sgRNA. Therefore, the effect of each off-target variant is small and could be treated as noises.

12. Despite the authors found sgRNAs were significantly depleted at day 10 in this study, other reported studies that used CRISPR-Cas9/sgRNA libraries for screening were often transduced about 14 days (PMID: 24336571, PMID: 28933431), 20 days (PMID: 27980086) or even four weeks (PMID: 31727847). The authors may need to re-evaluate their study by sequencing sgRNAs at extended time points, such as day 14 or longer.

We agree with the Reviewer that longer screen time may help to increase the depletion level of the sgRNAs that could generate deleterious variants. Our choice of 10 days after editing as our sequencing point were based on previous studies. CRISPR-Cas9 knockout screens were often performed after 14 doubling of cells after transduction [7]. From the growth assay, we found that the doubling time of eHAP cells is about 18 hours, which means that 10 days will approximately equal to the time for generating 14 doublings of eHAP cells. Also, a previous gene essentiality screen in HAP1 cells were also performed 10 days after transduction [3] .

1.	Arbab, M., et al., *Determinants of Base Editing Outcomes from Target Library Analysis and Machine Learning.* Cell, 2020.

2.	Landrum, M.J., et al., *ClinVar: public archive of relationships among sequence variation and human phenotype.* Nucleic Acids Res, 2014. **42**(Database issue): p. D980-5.

3.	Blomen, V.A., et al., *Gene essentiality and synthetic lethality in haploid human cells.* Science, 2015. **350**(6264): p. 1092-6.

4.	Daley, T.P., et al., *CRISPhieRmix: a hierarchical mixture model for CRISPR pooled screens.* Genome Biol, 2018. **19**(1): p. 159.

5.	Anzalone, A.V., L.W. Koblan, and D.R. Liu, *Genome editing with CRISPR–Cas nucleases, base editors, transposases and prime editors.* Nature Biotechnology, 2020. **38**(7): p. 824-844.

6.	Jin, S., et al., *Cytosine, but not adenine, base editors induce genome-wide off-target mutations in rice.* Science, 2019. **364**(6437): p. 292-295.

7.	Wang, T., E.S. Lander, and D.M. Sabatini, *Viral Packaging and Cell Culture for CRISPR-Based Screens.* Cold Spring Harb Protoc, 2016. **2016**(3): p. pdb prot090811.

**Second round of review**

**Reviewer 2**

I have read the revised version of "Identification of pathogenic variants in cancer genes using base editing screens with editing efficiency correction" and the authors response to the reviewers comment. Overall, I am satisfied with the changes made in the manuscript. I am especially happy that the authors took steps to make their code available online to help with reproducibility and increase transparency regarding statistical analysis. I also appreciate the authors' efforts in performing additional validation experiments.

However, based on the author's response, I am not sure that the gaussian mixture model was used in an appropriate fashion. If different models perform equally well, choosing one based on prior assumptions seems to be more confirmation bias than anything. Based on the data in Supp table 7, the 4 nuclei model never has optimal AIC nor BIC. I would also add that a change of -2 in AIC is generally considered a significant improvement. Based on this threshold, the only dataset for which the top ranked model (n=5) cannot be separated from the second best model (n=3) is the BRCA2_CG_NGG dataset. As such, I disagree with the authors statement that the models performed equally well. Additionally, data on the models is only provided for the NGG screens and not the NG screens. It should be available for both.

I think this aspect of the data analysis need reworking and suggest that the authors try another approach to categorize the scores, or at least make the multiple limitations of the current approach much, much clearer in the text. At the least, a clear statement of the FDR of deleterious variant in each screen should be presented.

**Reviewer 3**

In the revised manuscript entitled “Identification of pathogenic variants in cancer genes using base editing screens with editing efficiency correction”, Huang et al. have significantly improved their work by adding more descriptions, analyses and experimental data. However, some previous concerns are still failed to be fully addressed, as detailed below.

1. As for previous Q1, the authors have demonstrated that 74% of 729 variations overlapped between NGG and NG libraries were concordant in functional classification; however, the ratio of overlapped LOFs is far less than that of overall overlapped variants, as 35 LOF variants out of 67 NG-only ones (52%) and 35 LOF variants out of 125 NGG-only ones (28%). In this case, the concern is still not fully addressed, and it seems like that the screens by two different sets of libraries with different BEs are not comparable.

It is also strange that the authors thought their choice of positive controls was not a direct line of evidence to confirm the reliability of their screens. They explained that it was due to different degrees of essentiality of these genes in HAP1 cells and different PAM sequences for sgRNA design. If it were the case, did those 35 overlapped LOF variants found both in NG and NGG screens have the same PAM sequences for sgRNA design?

In addition, if not all “positive controls” could be screened out due to the inefficiency of NG library, what were functional scores of three sites (Q1200*, Y1509Y, Y1703C) that have been validated by AncBE4max in Line 153-156?

2. As for previous Q7, the authors revised their equation and directly denoted 𝑑𝑖𝑗 = 𝑞𝑖𝛽𝑖𝑗 due to the observation of an inverse linear relationship between ln fold change and the relative efficiency of nonsense variant production. However, Pearson’s correlation coefficient is only -0.507 and the tendency in Fig. 3b is not obvious as an inverse linear relationship. Is it possible that other non-linear relationships are more appropriate for this correlation?

It is not clear how the authors chose a 𝛽 score cutoff to define LOF variants (Figs. 4a-d, 5c-f). In line 284-286, they stated that “we classified the variants as loss-of-function if they have a higher probability belonging to the two negative-mean distributions than other distributions”, however, in line 286, “at a probability cutoff of 0.8, 126 and 290 variants were identified as loss-of-functions for BRCA1 and BRCA2”. What does a probability cutoff mean? It should be clearly addressed how to select a 𝛽 score cutoff to define LOF variants.

3. As for previous Q8, the authors have shown the correlation of ln fold change between BRCA2-CG-NGG and BRCA1-CG-NGG in Fig. S2e and the ln fold change distribution differences between positive and negative controls in Fig. S2f; however, it is still not clear how they calculated the distribution of functional scores (𝛽 score). It should be clarified.

4. As for previous Q10, if it were the case that the authors “did not use the HEK293T cells for base editing screen”, it is better to perform exogenous vector experiment in eHAP, but not HEK293T, cells. It seems like that correlations between the endogenous editing efficiencies in eHAP cells and those of integrated target sequences in HEK293T cells were not high (R=0.445 and 0.531, respectively) with either AncBE4max or ABEmax. It may be caused by using different cell lines. IN this case, to keep their study consistence, better to perform exogenous vector experiment in eHAP cells.

5. As for previous Q11, the authors excluded sgRNA dependent off-target effects from the final analysis and treated sgRNA independent off-targets as random noises. However, it is not clear why the authors chose these strategies to deal with off-target effects. For sgRNA dependent off-target effects, why excluded sgRNAs with three, but not two, target sites as off-targets? what was the criteria to define three target sites, with or without mismatch, how many mismatches? For sgRNA-independent off-targets, it has been reported that cytosine base editors generate substantial off-target single-nucleotide variants at both DNA and RNA levels (Zuo et al., Science 2019; Grunewald et al., Nature 2019; Zhou et al., Nature 2019). It is better not to treat them simply as noises.

6. As for previous Q2 by Review 1, the authors have now shown results of 7 randomly selected LOF sites in Fig. 4h, but failed to include the results of “control sgRNAs that targeted nonsense mutations … to evaluate the accuracy of this method”.

7. As for previous Q6 by Review 1, the authors now showed that filtering out sgRNAs with low editing efficiencies could increase the estimated sensitivity from 46.9% to 51.2% in NGG screen, which means setting higher editing efficiency cutoffs could further increase the sensitivity. Any comments on this?

**Authors Response**

**Point-by-point responses to the reviewers’ comments:**

Reviewer #2: I have read the revised version of “Identification of pathogenic variants in cancer genes using base editing screens with editing efficiency correction” and the authors response to the reviewers comment. Overall, I am satisfied with the changes made in the manuscript. I am especially happy that the authors took steps to make their code available online to help with reproducibility and increase transparency regarding statistical analysis. I also appreciate the authors’ efforts in performing additional validation experiments. However, based on the author’s response, I am not sure that the gaussian mixture model was used in an appropriate fashion. If different models perform equally well, choosing one based on prior assumptions seems to be more confirmation bias than anything. Based on the data in Supp table 7, the 4 nuclei model never has optimal AIC nor BIC. I would also add that a change of -2 in AIC is generally considered a significant improvement. Based on this threshold, the only dataset for which the top ranked model (n=5) cannot be separated from the second best model (n=3) is the BRCA2_CG_NGG dataset. As such, I disagree with the authors statement that the models performed equally well. Additionally, data on the models is only provided for the NGG screens and not the NG screens. It should be available for both. I think this aspect of the data analysis need reworking and suggest that the authors try another approach to categorize the scores, or at least make the multiple limitations of the current approach much, much clearer in the text. At the least, a clear statement of the FDR of deleterious variant in each screen should be presented.

We appreciate the reviewer’s positive feedback on our first-round response. For the concern on the method to classify the deleterious variants, we agree with the reviewer that the four-component model was not the best fit for some of the screen data based on AIC or BIC values. Instead, the 4-component model was selected based on its performance of classifying known LOF variants.

For both *NGG* and *NG* screens, we found that the 4-component model had the best AUC values on performance test in comparison to other Gaussian mixture models for all the screens (see the table below), using 49 “pathogenic/likely pathogenic” variants annotated in ClinVar as true positives and 190 “benign/likely benign” variants as true negatives. Based on AUC values, we also found that the best criteria to classify the loss-of-function (LOF) variants was to use the probability that a variant $i$ belonging to any of the negative-mean distributions ($P_{i}$) greater than 0.8 for NGG screens and $P_{i}$>0.9 for NG screens.

| **Library** | **Model name** | **Number of Gaussian Kernels for each library** | | **Probability that a variant belonging to any of the distribution with negative means** | | | | |
| --- | --- | --- | --- | --- | --- | --- | --- | --- |
|  |  |  |  | **>=0.7** | **>=0.75** | **>=0.8** | **>=0.85** | **>=0.9** |
| NGG | GMM_AIC-best | BRCA1_AT_ NGG: | 3 | 0.61 | 0.62 | 0.62 | 0.65 | 0.63 |
|  |  | BRCA1_CG_NGG: | 2 |  |  |  |  |  |
|  |  | BRCA2_AT_ NGG: | 5 |  |  |  |  |  |
|  |  | BRCA2_CG_NGG: | 5 |  |  |  |  |  |
|  | GMM_2n | All: | 2 | 0.58 | 0.61 | 0.6 | 0.6 | 0.58 |
|  | GMM_3n | All: | 3 | 0.6 | 0.63 | 0.63 | 0.62 | 0.58 |
|  | GMM_4n | All: | 4 | 0.61 | 0.66 | 0.66 | 0.65 | 0.62 |
|  | GMM_5n | All: | 5 | 0.62 | 0.63 | 0.65 | 0.64 | 0.64 |
| NG | GMM_AIC-best | BRCA1_AT_ NGG: | 2 | 0.55 | 0.54 | 0.53 | 0.53 | 0.52 |
|  |  | BRCA1_CG_NGG: | 2 |  |  |  |  |  |
|  |  | BRCA2_AT_ NGG: | 2 |  |  |  |  |  |
|  |  | BRCA2_CG_NGG: | 2 |  |  |  |  |  |
|  | GMM_2n | All: | 2 | 0.55 | 0.54 | 0.53 | 0.53 | 0.52 |
|  | GMM_3n | All: | 3 | 0.59 | 0.57 | 0.58 | 0.57 | 0.59 |
|  | GMM_4n | All: | 4 | 0.55 | 0.55 | 0.58 | 0.58 | 0.6 |
|  | GMM_5n | All: | 5 | 0.51 | 0.53 | 0.53 | 0.55 | 0.53 |

Table 1 for review: AUC values of different Gaussian mixture models for classifying loss-of-function variants (added as Additional file2: table S11 in manuscript).

For estimating the FDR, we first calculated the p-values using the Wald test against the null hypothesis that a variant having a $\beta$ score equal to or more than 0. Based on the p-values, we calculated the false discovery rate (FDR) for each variant using the Benjamini & Hochberg (BH) method. The FDR was under 0.2 for all the identified LOF variants in the NGG screen. For the NG screen, with P_i_ > 0.9 as the criteria for LOF variants, the FDR was under 0.1. We thus estimated that our FDR of deleterious variant was 0.2 for the NGG screen and 0.1 for the NG screen.

As suggested, we have tried to categorize LOF variants using $\beta$ score cutoffs.

However, the performance of the uniform $\beta$ score method was worse than the Gaussian mixture model method (AUC=0.6 vs. 0.66 for NGG screens and AUC=0.56 vs. 0.6 for NG screens). Because the score distributions for four libraries were different, a uniform $\beta$ score cutoff may not apply for all screens to identify LOF. In our 4-component Gaussian mixture model, with the criteria P_i_ > 0.8 for all NGG screens, the equivalent $\beta$ score cutoffs for LOF variants were -0.55, -0.45, -0.37 and -0.4 in *BRCA1_AT_NGG*, *BRCA1_CG_NGG*, *BRCA2_AT_NGG* and *BRCA2_CG_NGG*, respectively.

Therefore, using Gaussian mixture modeling, we were able to use a uniform probability cutoff across different screens to achieve the best classification of LOF variants, while controlling the false discovery rates to an acceptable range.

We added a **Supplementary Note 1** to describe the selection of Gaussian mixture model based on AUC values. The codes for model selection are available at Github (https://github.com/guangyu1990/Edit_Efficiency_Correction). The AIC/BIC values for NGG and NG screens were now listed in Supplementary Table S10. And the AUC values of different models were added in the new supplementary Table S11. The p-values from Wald test and the FDR are now listed in the column “Wald-P values” and “Wald-FDR” in supplementary Table S6. We also modified the main text to reflect the changes:

“Using the efficiency-correction model, for each sgRNA, we calculated the functional score for the major outcome generated by that sgRNA (**Fig. 3a; Additional file 2: Table S6**). To identify loss-of-function variants, we chose the mixture model of four Gaussian components to fit the score distributions for each screen (**Supplementary Note 1**). For each library, the majority of scores followed a normal distribution with a mean at about zero, consistent with our assumption that majority of variants with no impact on *BRCA1/2* function. The two subpopulations following distributions with negative means were likely corresponding to the variants that having deleterious effects on gene function (**Additional file 1: Fig. S5a-d**). We classified the variants as “loss-of-function” if their probability belonging to any of the two negative-mean distributions were more than 0.8 (See methods “Identification of loss-of-function variants” and Supplementary Note 1). The equivalent $\beta$ score cutoffs were -0.55, -0.45, -0.37 and -0.4 for *BRCA1_AT_NGG*, *BRCA1_CG_NGG*, *BRCA2_AT_NGG* and *BRCA2_CG_NGG* libraries, respectively. In total, 121 and 290 variants were identified as loss-of-function mutations for *BRCA1* and *BRCA2*, respectively (**Fig. 4a-d**). We estimated that the FDR for LOF variants was at a level of 0.2 (Supplementary Note 1).”

”

Reviewer #3: In the revised manuscript entitled “Identification of pathogenic variants in cancer genes using base editing screens with editing efficiency correction”, Huang et al. have significantly improved their work by adding more descriptions, analyses and experimental data. However, some previous concerns are still failed to be fully addressed, as detailed below.

1. As for previous Q1, the authors have demonstrated that 74% of 729 variations overlapped between NGG and NG libraries were concordant in functional classification; however, the ratio of overlapped LOFs is far less than that of overall overlapped variants, as 35 LOF variants out of 67 NG-only ones (52%) and 35 LOF variants out of 125 NGG-only ones (28%). In this case, the concern is still not fully addressed, and it seems like that the screens by two different sets of libraries with different BEs are not comparable. It is also strange that the authors thought their choice of positive controls was not a direct line of evidence to confirm the reliability of their screens. They explained that it was due to different degrees of essentiality of these genes in HAP1 cells and different PAM sequences for sgRNA design. If it were the case, did those 35 overlapped LOF variants found both in NG and NGG screens have the same PAM sequences for sgRNA design? In addition, if not all “positive controls” could be screened out due to the inefficiency of NG library, what were functional scores of three sites (Q1200*, Y1509Y, Y1703C) that have been validated by AncBE4max in Line 153-156?

We thank the reviewer for the careful analysis of our previous response and constructive suggestions. We apologize for some confusing descriptions we had in the previous version of manuscript.

First, we want to clarify that we evaluated the performance of our screen using all the variants in *BRCA1/2* that have been annotated in ClinVar database. We estimated the sensitivity as the percent of “Pathogenic/Likely pathogenic” variants that have been identified as LOF variants in our screen. And the specificity as the percent of “Benign/Likely benign” variants that have been identified as non-LOF variants. In our datasets, the sensitivity was 46.9% for the NGG screen and 21.2% for the NG screen. The specificity was 81.6% for the NGG screens and 88.9% for the NG screen.

In comparison to CRISPR-Cas9 knockout screen for essential genes (~70-80% sensitivity and 97-99% specificity [1, 2]), the sensitivity and specificity of our screen was significantly lower. We think that is mainly due to the limitation of current version of base editors. Base editors have at least 2 fold lower efficiencies in genome editing comparing to the efficiency of Cas9 to generate cleavage events [3-5]. In addition, for most variants, only one sgRNA could be designed to target each variant in each screen. In contrast, multiple sgRNAs with different target sequences were used in the CRISPR-Cas9 knockout system for one gene to increase the power of detection. In fact, it has been shown in Daley et al., for CRISPRi screens, when only one sgRNA per gene is used, they estimated the sensitivity to identify essential genes to be around 0.2 [1], i.e. only 20% true positives could be identified in CRISPRi screens.

The overlap ratio of LOF variants we observed for NG and NGG screens were consistent with the sensitivity and specificity we observed for NG and NGG screens. Assuming the two screens each sampled some true positives from the same pool with the given sensitivity and specificity of our screens, we simulated that the median overlap ratio was 25% for NG and 15% for NGG. This is consistent with our observation, which was 32.6% for NG and 20.9% for NGG (see the number of variants in the Table 2 for review). The total number of variants slightly changed from previous version, because we removed the sgRNAs that targeting more than one sites in the genome for the analysis.

|  | NG screen LOF | NG screen non-LOF | Total |
| --- | --- | --- | --- |
| NGG screen LOF | 32 | 121 | 153 |
| NGG screen non-LOF | 66 | 494 | 560 |
| Total | 98 | 615 | 713 |

Table 2 for review: Numbers of different categories of variants overlapped in NG and NGG screens (added as Additional file2: table S8 in manuscript).

We found that the overlap ratio will increase if the sensitivity of NG and NGG screen increases proportionally (Figure 1 for review, the specificities were not changed for both screens). The low sensitivity and specificity of the screen is likely the major cause for the low overlap ratio we have observed. Indeed, for CRISPR-Cas9 knockout screens to identify essential genes, when the sensitivities were as 0.79 and 0.82 for two screens, and specificities were as high as 0.99 and 0.97 for two screens, their simulated overlap ratio were at a median of 73% and 60%. This is also in agreement with the observed overlap ratio of 81% and 67% for the DepMap data [6].

Figure 1 for review. Simulated overlap ratios of NG and NGG screens. The X-axis is the sensitivity of NGG screen. The NG screen sensitivity is simulated as 2.2 fold less of the NGG screen. The specificity was kept as 0.82 and 0.89 for the NGG screens and NG screens, respectively.

For the three variants that have been validated by AncBE4max, Y1509Y was not included in the NG library. Q1200* and Y1703C were included and not classified as LOF variants in NG screen. In NG screen, the fold change of sgRNAs targeting Q1200* and Y1703 were 0.932 and 0.971 and the functional scores were -0.156 and -0.254, respectively. Such low signals of the two variants are likely due to the inefficiency of “NG” recognizing base editors in comparison to the “NGG” recognizing base editors.

We now added the following sentence in the discussion when addressing the multiple limitations of current version of BE screen (paragraph 1, page 21):

“Further, only one sgRNA was used for each variant, which could lead to low sensitivity for identifying LOF variants. For the CRISPRi screens, when only one sgRNA was used, it has been estimated that the sensitivity for identifying essential genes to be less than 0.2 [38].”

We also added the table of number of overlaps with NG and NGG screens in Supplementary Table S8 for clarity.

2. As for previous Q7, the authors revised their equation and directly denoted = due to the observation of an inverse linear relationship between ln fold change and the relative efficiency of nonsense variant production. However, Pearson’s correlation coefficient is only -0.507 and the tendency in Fig. 3b is not obvious as an inverse linear relationship. Is it possible that other non-linear relationships are more appropriate for this correlation? It is not clear how the authors chose a score cutoff to define LOF variants (Figs. 4a-d, 5c-f). In line 284-286, they stated that “we classified the variants as loss-of-function if they have a higher probability belonging to the two negative-mean distributions than other distributions”, however, in line 286, “at a probability cutoff of 0.8, 126 and 290 variants were identified as loss-of-functions for BRCA1 and BRCA2”. What does a probability cutoff mean? It should be clearly addressed how to select a score cutoff to define LOF variants.

We agree that it is possible that other nonlinear relationships are more appropriate for this correlation. However, given the small number of points in Fig. 3b, it is very difficult to find the best nonlinear model for the data without over-fitting. Also, from the data in Fig. 3c, which also represented the relationship between ln fold change and editing efficiencies, were consistent with the linear model (r=-0.554, p=0.003). Therefore, we think a linear model is a safe choice at our situation.

We also tried to use the exponential cell growth model we described in the Methods section “modeling sgRNA functional scores with editing efficiency”, i.e. the formula $d_{ij}={ln(1+q}_{i}\beta_{ij})$ to calculate the functional scores β. The scores were highly correlated with the linear score (Pearson’s r=0.983, p-value=0.0000012). Using these scores for LOF classification, the AUC values were almost the same as using the linear model scores (0.66 for NGG screen and 0.58 for NG screen).

We included in the discussion, that the linear model may not apply to all BE screens (paragraph 1, page 20):

“It is noted that although efficiency-correction is needed for better identification of loss-of-function variants in all BE screen experiments, the linear relationship between ln fold change and editing efficiency may not directly apply for other base editing screens. Other efficiency-correction models may be developed under our framework to improve variant classification.”

For identifying LOF variants, we fitted four-component Gaussian mixture models on the functional score distribution of all the variants that targeting BRCA1/2 in each screen, as shown in Figure S5. The model treated the data as a mixture of four Gaussian distributions with different means, two of them had negative mean values. We then use the probability that the $\beta$ score belonging to any of the distribution with negative means in the Gaussian mixture model to define the LOF variants. We used the probability of 0.8 as the cutoff for all the NGG screen libraries, the equivalent $\beta$ score cutoffs were -0.55, -0.45, -0.37 and -0.4 for *BRCA1_AT_NGG*, *BRCA1_CG_NGG*, *BRCA2_AT_NGG* and *BRCA2_CG_NGG* libraries, respectively. Because the score distribution for four libraries were different, using the probability cutoffs to control false discovery rate avoids the arbitrary selection of $\beta$ score cutoff for each screen.

We change the description of variants classification and describe the equivalent score cutoffs in the text (paragraph 3, page 12).

“To identify loss-of-function variants, we chose the model of four Gaussian components to fit the score distributions (**Supplementary Note 1**). For each library, the majority of scores followed a normal distribution with a mean at about zero, consistent with our assumption that majority of variants with no impact on *BRCA1/2* function. The two subpopulations following distributions with negative means were likely corresponding to the variants that having deleterious effects on gene function (**Additional file 1: Fig. S5a-d**). We classified the variants as “loss-of-function” if their probability belonging to any of the two negative-mean distributions were more than 0.8 (See methods “Identification of loss-of-function variants” and Supplementary Note 1). The equivalent $\beta$ score cutoffs were -0.55, -0.45, -0.37 and -0.4 for *BRCA1_AT_NGG*, *BRCA1_CG_NGG*, *BRCA2_AT_NGG* and *BRCA2_CG_NGG* libraries, respectively. In total, 121 and 290 variants were identified as loss-of-function mutations for *BRCA1* and *BRCA2*, respectively (**Fig. 4a-d**). We estimated that the FDR for LOF variants was at a level of 0.2 (Supplementary Note 1).”

3. As for previous Q8, the authors have shown the correlation of ln fold change between BRCA2-CG-NGG and BRCA1-CG-NGG in Fig. S2e and the ln fold change distribution differences between positive and negative controls in Fig. S2f; however, it is still not clear how they calculated the distribution of functional scores ( score). It should be clarified.

As shown in the answer to Q2, we identified loss-of-function variants by fitting the distribution of functional scores of all the variants in each screen using a four-component Gaussian mixture model.

The so-called “positive controls”, which were a group of sgRNAs that may generate stop gain mutations on essential genes other than *BRCA1/2*, were not used to calculate the distribution of functional scores, nor for the selection of $\beta$ score cutoffs for identification of LOF variants. To avoid misunderstanding, we now referred to the previous “positive controls” as “essential gene controls”.

For clarity, the details about model selection and validation of LOF identification were now added in the **Supplementary Note 1**.

4. As for previous Q10, if it were the case that the authors “did not use the HEK293T cells for base editing screen”, it is better to perform exogenous vector experiment in eHAP, but not HEK293T, cells. It seems like that correlations between the endogenous editing efficiencies in eHAP cells and those of integrated target sequences in HEK293T cells were not high (R=0.445 and 0.531, respectively) with either AncBE4max or ABEmax. It may be caused by using different cell lines. IN this case, to keep their study consistence, better to perform exogenous vector experiment in eHAP cells.

We thank the reviewer for the suggestion. Yes, it is possible that the additional difference between endogenous editing efficiencies and those measured by integrated target assay are caused by using different cell lines.

We added the point in the text (paragraph 1, page 11):

“The consistency with endogenous editing efficiencies may be improved by performing the integrated target site assay in HAP1 cells.”

5. As for previous Q11, the authors excluded sgRNA dependent off-target effects from the final analysis and treated sgRNA independent off-targets as random noises. However, it is not clear why the authors chose these strategies to deal with off-target effects. For sgRNA dependent off-target effects, why excluded sgRNAs with three, but not two, target sites as off-targets? what was the criteria to define three target sites, with or without mismatch, how many mismatches? For sgRNA-independent off-targets, it has been reported that cytosine base editors generate substantial off-target single-nucleotide variants at both DNA and RNA levels (Zuo et al., Science 2019; Grunewald et al., Nature 2019; Zhou et al., Nature 2019). It is better not to treat them simply as noises.

We appreciate the reviewer for pointing out the issue of off-target effects. For sgRNA dependent off-target effects, we used Cas-OFFinder [7] to find the off-target sites in the genome without allowing mismatches. As suggested, we now excluded those sgRNAs with more than one target site in the genome in the analysis of screen data. We changed the text accordingly and added the Cas-OFFinder information in the method section ”Oligonucleotide-library design and synthesis” (paragraph 1, page 24):

“The off-target sites for each sgRNA were predicted using Cas-OFFinder [44], without mismatch in protospacer.”

For sgRNA independent off-target effects, we agree that it is a caveat of the current version of base editors. Because these off-target single-nucleotide variants by base editors without hotspots, they can be estimated from the distribution of fold changes of ~50 non-targeting sgRNAs in each screen library. By normalizing the ln fold change of sgRNAs to non-targeting control sgRNAs, we have taken such off-target effects into consideration in our screen analysis.

We added the off-target effects as a potential limitation of BE screens that could be addressed further in the future in the discussion:

“The off-target effects of base editors are also a potential concern[39-41]”.

6. As for previous Q2 by Review 1, the authors have now shown results of 7 randomly selected LOF sites in Fig. 4h, but failed to include the results of “control sgRNAs that targeted nonsense mutations … to evaluate the accuracy of this method”.

Thanks for pointing it out the oversight. The 7 LOF sites shown in last version of Fig. 4h included three nonsense mutations (Q3206*, Q1502*, and Q1327*). We now added the control sgRNAs (Q1200*, Y1509Y, Y1703C) in Fig. 1h to Fig. 4h. The fold change of sgRNAs for generated these variants in the screen correlated well with the degree of delayed growth (Pearson r=0.907, p=0.0003).

7. As for previous Q6 by Review 1, the authors now showed that filtering out sgRNAs with low editing efficiencies could increase the estimated sensitivity from 46.9% to 51.2% in NGG screen, which means setting higher editing efficiency cutoffs could further increase the sensitivity. Any comments on this?

As the reviewer has predicted, setting higher editing efficiency cutoffs further increased the sensitivity of the NGG screen. As shown in the table below, while the number of LOF variants detected reduces as the editing efficiency cutoff increases, the sensitivity increased to as high as 62.5% with the cutoff of 0.35. The results suggested that the fold changes of sgRNAs with higher editing efficiencies were more robustly measured in the screen. We use the editing efficiency cutoff of 0.1 in our analysis to maximize the number of LOF variants.

| Efficiency | Pathogenic variants identified as LOF /Total pathogenic variants | Sensitivity | Specificity | Total LOF variants |
| --- | --- | --- | --- | --- |
| 0.1 | 23\|49 | 0.469 | 0.812 | 411 |
| 0.15 | 19\|39 | 0.487 | 0.856 | 333 |
| 0.2 | 19\|37 | 0.514 | 0.877 | 256 |
| 0.25 | 15\|27 | 0.556 | 0.88 | 190 |
| 0.3 | 12\|21 | 0.571 | 0.885 | 131 |
| 0.35 | 10\|16 | 0.625 | 0.892 | 87 |
| 0.4 | 8\|14 | 0.571 | 0.931 | 64 |
| 0.45 | 7\|13 | 0.538 | 0.895 | 51 |
| 0.5 | 3\|9 | 0.333 | 0.9 | 34 |

1.	Daley, T.P., et al., *CRISPhieRmix: a hierarchical mixture model for CRISPR pooled screens.* Genome Biol, 2018. **19**(1): p. 159.

2.	Hart, T. and J. Moffat, *BAGEL: a computational framework for identifying essential genes from pooled library screens.* BMC Bioinformatics, 2016. **17**: p. 164.

3.	Arbab, M., et al., *Determinants of Base Editing Outcomes from Target Library Analysis and Machine Learning.* Cell, 2020. **182**(2): p. 463-480 e30.

4.	Song, M., et al., *Sequence-specific prediction of the efficiencies of adenine and cytosine base editors.* Nat Biotechnol, 2020. **38**(9): p. 1037-1043.

5.	Kim, H.K., et al., *High-throughput analysis of the activities of xCas9, SpCas9-NG and SpCas9 at matched and mismatched target sequences in human cells.* Nat Biomed Eng, 2020. **4**(1): p. 111-124.

6.	Dempster, J.M., et al., *Agreement between two large pan-cancer CRISPR-Cas9 gene dependency data sets.* Nat Commun, 2019. **10**(1): p. 5817.

7.	Bae, S., J. Park, and J.S. Kim, *Cas-OFFinder: a fast and versatile algorithm that searches for potential off-target sites of Cas9 RNA-guided endonucleases.* Bioinformatics, 2014. **30**(10): p. 1473-5.

**Third round of review**

**Reviewer 2**

The supplementary note added by the authors addresses my previous concerns, and I thank the authors for making their methods clearer. My only comment on the revised manuscript is that I couldn't find mention of the FDR for variants identified in the NG screen in the main text when its results are discussed.

**Reviewer 3**

In the revised manuscript entitled “Identification of pathogenic variants in cancer genes using base editing screens with editing efficiency correction”, Huang et al., have further improved their study by addressing all my previous concerns, except one. As for their response to the previous Q3, the authors have stated “ The so-called “positive controls”, which were a group of sgRNAs that may generate stop gain mutations on essential genes other than BRCA1/2, were not used to calculate the distribution of functional scores, nor for the selection of β score cutoffs for identification of LOF variants”. However, if they were true “positive controls”, it is not clear why functional scores of these so-called “positive controls” were not calculated or included in the distributions of all functional scores? Please further explain. Other than this concern, I am satisfied with the current version of this manuscript.

**Authors Response**

**Point-by-point responses to the reviewers’ comments:**

Reviewer #2: The supplementary note added by the authors addresses my previous

concerns, and I thank the authors for making their methods clearer. My only comment

on the revised manuscript is that I couldn't find mention of the FDR for variants

identified in the NG screen in the main text when its results are discussed.

As suggested, we added the information in the main text:

“After filtering for the sgRNAs that have low editing activities (scaled activity less than

0.2), we associated 567 variants with negative effects on the function of BRCA1/2 (Fig.

5c-f), the estimated FDR for these variants were under 0.1 (Additional file 3:

Supplementary Note 1).”

Reviewer #3: In the revised manuscript entitled “Identification of pathogenic variants in

cancer genes using base editing screens with editing efficiency correction”, Huang et

al., have further improved their study by addressing all my previous concerns, except

one. As for their response to the previous Q3, the authors have stated “The so-called

“positive controls”, which were a group of sgRNAs that may generate stop gain

mutations on essential genes other than BRCA1/2, were not used to calculate the

distribution of functional scores, nor for the selection of β score cutoffs for identification

of LOF variants”. However, if they were true “positive controls”, it is not clear why

functional scores of these so-called “positive controls” were not calculated or included

in the distributions of all functional scores? Please further explain. Other than this

concern, I am satisfied with the current version of this manuscript.

Those so-called “positive controls”, i.e., the sgRNAs that may generate stop gain

mutations on essential genes other than BRCA1/2, were positive controls for cell

lethality in the screen, but not positive controls for identifying loss-of-function variants in

BRCA1/2. Instead, we used known pathogenic variants in BRCA1/2 as true positive

controls for the classification of BRCA1/2 variants using efficiency-corrected functional

scores. Therefore, we did not include the scores of these so-called “positive controls”

for calculating the functional score distribution in each screen.

For clarity, we referred these sgRNAs as “essential gene controls” and modified the

sentence to the following one when mentioning these sgRNAs:

“For BRCA1-CG-NGG and BRCA2-CG-NGG libraries, we also included 230 sgRNAs

that could generate stop codon in essential genes other than BRCA1/2 as additional

controls for cell lethality in the screen (hereafter referred as “essential gene controls”).”
